# Brain injury induces HIF-1α-dependent transcriptional activation of LRRK2 that exacerbates brain damage

**DOI:** 10.1038/s41419-018-1180-y

**Published:** 2018-11-12

**Authors:** Yun-Hee Bae, Hyejin Joo, Jinhyun Bae, Seung Jae Hyeon, Song Her, Eunhwa Ko, Hwan Geun Choi, Hoon Ryu, Eun-Mi Hur, Youngmin Bu, Byoung Dae Lee

**Affiliations:** 1 Department of Neuroscience, Graduate School, Kyung Hee University, Seoul, 02447 Republic of Korea; 2 Department of Science in Korean medicine, Graduate School, Kyung Hee University, Seoul, 02447 Republic of Korea; 3 Department of Herbal Pharmacology, College of Korean Medicine, Kyung Hee University, Seoul, 02447 Republic of Korea; 4 Center for Neuroscience, Brain Science Institute, Korea Institute of Science and Technology, Seoul, 02792 Republic of Korea; 5 Korea Basic Science Institute, Seoul, 03759 Republic of Korea; 6 New Drug development center, Daegu-Gyeongbuk Medical Innovation Foundation, Daegu, 41061 Republic of Korea; 7 Veteran’s Affairs Boston Healthcare System, Boston, MA 02130 USA; 8 Boston University Alzheimer’s Disease Center and Department of Neurology, Boston University School of Medicine, Boston, MA 02118 USA; 9 Department of Neuroscience, College of Veterinary Medicine, Research Institute for Veterinary Science, and BK21 PLUS Program for Creative Veterinary Science Research, Seoul National University, Seoul, 08826 Republic of Korea; 10 Department of Physiology, Kyung Hee University School of Medicine, Seoul, 02447 Republic of Korea

## Abstract

*Leucine-rich repeat kinase 2* (*LRRK2*), originally identified as a causative genetic factor in Parkinson’s disease, is now associated with a number of pathologies. Here, we show that brain injury induces a robust expression of endogenous LRRK2 and suggest a role of LRRK2 after injury. We found that various in vitro and in vivo models of traumatic brain injury (TBI) markedly enhanced LRRK2 expression in neurons and also increased the level of hypoxia-inducible factor (HIF)-1α. Luciferase reporter assay and chromatin immunoprecipitation revealed direct binding of HIF-1α in LRRK2 proximal promoter. We also found that HIF-1α-dependent transcriptional induction of *LRRK2* exacerbated neuronal cell death following injury. Furthermore, application of G1023, a specific, brain-permeable inhibitor of LRRK2, substantially prevented brain tissue damage, cell death, and inflammatory response and alleviated motor and cognitive defects induced by controlled cortical impact injury. Together, these results suggest HIF-1α-LRRK2 axis as a potential therapeutic target for brain injury.

## Introduction

Leucine-rich repeat kinase 2 (LRRK2) is a large protein comprising the GTPase and the kinase domains, surrounded by multiple protein-protein interacting domains. In the brain, LRRK2 is expressed in various regions including the cortex, hippocampus, striatum, and substantia nigra pars compacta^1,2^. *LRRK2* is associated with both familial and sporadic Parkinson’s disease (PD)^3–5^. Mutations in PD cluster within the two catalytic domains and ectopic expression of the hyperactive, pathogenic LRRK2-G2019S mutant induces cell death^6–9^, suggesting that aberrantly increased kinase activity is associated with neurotoxicity and the progression of PD. Consistent with the presence of multiple, enzymatic and protein-interacting domains, LRRK2 mutants interfere with diverse cellular processes, including vesicular trafficking^1,10,11^, cytoskeleton dynamics^12^, protein translation^13^, autophagy^14,15^, and mitochondria functions^16^. Although significant progress has been made towards our understanding of the pathogenic mutants of LRRK2, physiological functions of the endogenous, wild-type LRRK2 still remains largely ambiguous.

In addition to neurons, LRRK2 is expressed in immune cells^17,18^, and LRRK2 has been identified as one of the susceptibility genes for leprosy and Crohn’s disease^19,20^. In various types of peripheral immune cells, LRRK2 expression has been shown to be upregulated by proinflammatory signals, such as interferon (IFN)-γ, interleukin (IL)-1β, and lipopolysaccharide (LPS)^17,18,21,22^. In microglia, LRRK2 expression could be induced by LPS both in vivo^23^ and in vitro^24^, and pharmacological inhibition or depletion of LRRK2 could attenuate LPS-induced proinflammatory signals, such as secretion of tumor necrosis factor (TNF)-α and induction of nitric oxide synthase^23^. These studies suggest that immune cells increase the level of endogenous LRRK2 and that LRRK2 may play a modulatory role in inflammatory responses. However, little is known about the mechanism of LRRK2 induction, and it remains to be determined if endogenous LRRK2 level is also regulated in other cell types.

Traumatic brain injury (TBI) refers to an insult to the brain from an external mechanical force^25–27^ and is the leading cause of morbidity and mortality in individuals under the age of 45^28^. The survivors of TBI are left with significant long-term sequelae, such as physical and cognitive deficits and changes in personality. TBI is initiated by primary brain injury and followed by delayed and protracted secondary injury, which is often more damaging than the primary injury. The secondary injury process involves diverse pathological mechanisms, including hypoxic-ischemic injury, excitotoxicity, oxidative stress, and chronic inflammation^29–31^. Since secondary injury occurs over time, it presents an amendable therapeutic window to treat TBI, but efforts to develop effective intervention have fallen short.

Using various cellular and in vivo models of injuries, here we found that neural injury activates hypoxia induced factor (HIF) 1α-dependent transcriptional regulatory mechanism to induce the expression of endogenous LRRK2 in neurons. We also observed an increase in the mRNA and protein levels of LRRK2 in postmortem brains of chronic traumatic encephalopathy (CTE) patients. Furthermore, pharmacological inhibitors of LRRK2 not only ameliorated neuronal cell death and neuroinflammation but also prevented the behavioral defects in mice caused by controlled cortical impact (CCI) injury. Together, our results suggest that LRRK2 functions to exacerbate neuronal cell death after brain injury and that LRRK2 might be a potential therapeutic target for brain injury.

## Materials and methods

### Chemicals and antibodies

Cobalt(II) chloride hexahydrate (CoCl_2_), L-Glutamic acid monosodium salt hydrate, hydrogen peroxide (H_2_O_2_), and 2-Methoxyestradiol (2ME2) were purchased from Sigma-Aldrich. GSK2578215A (#4629) and LRRK2-IN-1 (#4273) were obtained from Tocris Bioscience. G1023, MLi-2, and PF-06447475 were synthesized by Dr. Hwan Geun Choi at Daegu-Gyeongbuk Medical Innovation Foundation (DGMIF, Daegu, South Korea). Information about antibodies used in this study is provided in Supplementary Table 2.

### Animals

Pregnant and adult ICR mice were purchased from DBL. ICR mice were acclimatized for one week under conditions of controlled temperature (22 ± 2 °C), constant humidity, and a 12 h light/dark cycle, and food and water were made available ad libitum. All animal procedures were approved by the Institutional Animal Care and Use Committee of Kyung Hee University (KHUASP(SE)-17-079).

### Mouse controlled cortical impact injury model

Mice were randomly assigned to each experimental groups. In vivo model of TBI was induced using the CCI method^32,33^. Briefly, ICR mice (8 weeks old, male, 30–33 g) were anesthetized with 2% isoflurane in gas mixture of 70% N_2_O and 30% O_2_, and placed in the stereotaxic frame. After a midline incision of the scalp, circular craniotomy was performed using an electric drill with a trephine bit (4-mm in diameter) on the right parietal cortex (anterior-posterior (AP)- 2 mm, medial-lateral (ML) 2.0 mm from bregma). CCI injuries were produced at an impact depth of 2.5 mm, with a 2-mm diameter round impact tip (velocity of 2 m/s, dwell time of 300 ms) and 25° angle to the dura meter, using an electromagnetically driven CCI injury device (Impact One^TM^ Stereotaxic Impactor for CCI, Leica Microsystems). The impact site was recovered by the skull which was isolated before impacting and fixed by glue. Body temperature was monitored by a rectal thermometer during surgery and was controlled at 37 ± 0.5°C using a heating pad. Sham-operated animals underwent craniotomy without impact. Less than 5% of the injured mice died after surgery due to complication of the injury. None of the mice that survived were excluded post hoc.

### Brain preparation

For RT-PCR and western blot analysis, ipsilateral side of the CCI-injured brains were dissected around 4 mm from the margin of contusion site on ice, immediately frozen in dry ice, and stored at −80°C until use. For immunohistochemistry, the mice were anesthetized with an overdose pentobarbital (50 mg/kg, intraperitoneal injection) and transcardially perfused with PBS followed by 4% paraformaldehyde (w/v in PBS). Brains were harvested, post-fixed overnight in 4% PFA, then cryoprotected in 30% sucrose solution (w/v in PBS) and stored at 4°C. Serial 30 μm thick coronal sections were cut using a cryostat (Leica CM 1950) and stored in cryoprotectant solution (30% glycerol, 30% ethylene glycol, 40% PBS). The sections were then processed for histological analysis.

### Human tissues

Normal and CTE human brain samples were from the Boston University Alzheimer’s Disease Center (BUADC) and Chronic Traumatic Encephalopathy (CTE) Center. The study involves only tissue collected from postmortem, and consequently not classified as human subjects. Next of kin provided informed consent for participation and brain donation. The study was performed in accordance with institutional regulatory guidelines. Detailed information of brain tissues is described in Supplementary Table 1.

### Immunohistochemistry of human brain tissue

Paraffin-embedded postmortem brain tissues were sectioned in a coronal plane at 10–20 μm. The tissue sections were deparaffinized and rehydrated. Heat-mediated antigen retrieval was performed by submerging the slides in 10 mM citrate buffer (pH 6.0). Then, the sections were blocked with blocking solution (1% H_2_O_2_), and incubated with LRRK2 (abcam, ab133474) for 24 h. After three times of washing, the slides were processed with Vector ABC Kit (Vector Lab). The immunoreactive signals were developed with DAB chromogen (Thermo Fisher Scientific) and analyzed under a bright field microscopy

### Preparation of mouse primary cortical neurons

Primary cortical neurons were prepared from embryonic day 15.5 ICR mice as described previously^9^ with minor modifications. Cortices were dissected in HBSS (GIBCO), followed by digestion in papain (20 U/ml, Worthington Biochemical) and DNase (10 U/ml, Sigma-Aldrich) diluted in HBSS for 20 min at 37°C. Enzyme-digested cortices were washed three times with Minimum Essential Medium (MEM, GIBCO) containing 10% heat inactivated fetal bovine serum (FBS, GenDEPOT) and dissociated in culture medium. For RT-PCR and western blotting, dissociated neurons were plated at a density of 8 × 10^5^ cells per well into 6-well culture plates. For immunocytochemistry, cells were plated at a density of 2 × 10^5^ cells per well into 12-well culture plates containing 18-mm glass coverslips coated with 50 μg/ml poly-D-lysine (Sigma-Aldrich). After overnight incubation, media was changed to phenol red-free Neurobasal A (GIBCO) containing 1% Glutamax-I (GIBCO) and 2% B27 supplement (GIBCO) and cells were cultured at 37°C in a 5% CO_2_ incubator. Every 3 days, half of the culture medium was replaced with fresh medium.

### Scratch injury model

In vitro model of TBI was induced by scratch injury^34,35^. Briefly, confluent cultures were manually scratched using a sterile pipette tip (10 μl), which produced a linear teat across the culture well. In each well of the 6-well and 12-well culture plates, 11 × 11 scratches and 6 × 6 scratches were induced, respectively, producing a 3 mm grid. Cultures were incubated without change of medium. Scratches caused immediate death to cells directly contacting the tip, which followed by a progressive secondary injury to neurons located at a distance from the scratches. Non-scratched cells were used as control.

### RNA isolation and RT-PCR

Total RNA was isolated from mouse brain tissues or cells using Trizol reagent (Invitrogen). cDNA synthesis was performed using 2 μg of total RNA with a M-MLV reverse transcriptase kit (Promega) according to the manufacturer’s instructions. The sequences of PCR primers are shown in the Supplementary Table 3. The PCR products were separated by on a 1.5% agarose gel and band intensities were quantified using the Image J software (National Institute of Health).

### Quantitative real-time PCR

Total RNA was extracted from the frozen brain tissues by TRIzol reagent (MRC, TR118). Fifty nanograms of RNA were used as a template for quantitative RT-PCR amplification, using SYBR Green Real-time PCR Master Mix (Toyobo). Primers were standardized in the linear range of cycle before the onset of the plateau. Primer sequences of LRRK2 and GAPDH are given in Supplementary Table 3. GAPDH was used as an internal control. The real-time data acquisition was performed with an LightCyler96 Real-Time PCR System (Roche Diagnostics) using the following cycle conditions: 95℃ for 1 min x1 cycle, and 95℃ for 15 s, followed by 60℃ for 1 min x 45 cycles. The relative expression of gene was analyzed by the LightCyler96 software and expressed as Ct the number of cycles needed to generate a fluorescent signal above a predefined threshold.

### Western blot analysis

Brain tissues were homogenized and lysed with a glass dounce tissue grinder in NETN buffer (40 mM Tris-HCl (pH 7.4), 120 mM NaCl, 10 mM EDTA, 0.5% Nonidet P-40) containing protease inhibitors (2 μg/ml Aprotinin, 1 μg/ml Pepstatin, 1 μg/ml Leupeptin, 1 mM PMSF), phosphatase inhibitors (10 mM β-glycerophasphate, 50 mM NaF, 1 mM Na_3_VO_4_), and 1 mM DTT. Primary cortical neurons were lysed in the buffer described above. The homogenates were incubated for 30 min on ice and then centrifuged at 13,000 rpm for 20 min at 4°C. Protein concentration was determined with a BCA protein assay kit (Thermo Fisher Scientific). Equal amounts of protein were loaded and resolved on 6–12% SDS-PAGE gels and transferred to PVDF membranes. Membranes were blocked in 5% skim milk in PBS-T (0.1% Tween20 in PBS) for 1 h and then incubated overnight at 4°C with the primary antibodies. After washing three times with PBS-T, the membranes were incubated with appropriate secondary antibodies for 1 h at room temperature. Signals were developed using enhanced chemiluminescence (Millipore) and detected by exposure to X-ray film (AGFA). The band intensities were quantified by densitometry analysis using Image J software (National Institute of Health).

### Immunocytochemistry

Primary cortical neurons grown on coverslips were fixed in ice-cold methanol for 10 min on ice. Neurons were permeabilized and blocked in a solution containing 2% BSA, 0.2% Triton X-100 in PBS for 1 h. Primary antibodies were diluted in PBS containing 0.2% Triton X-100 and 0.5% BSA and secondary antibodies in PBS. Neurons were incubated with appropriate primary antibodies overnight at 4°C and then washed with PBS followed by incubation with Alexa 488 or Alexa 568-conjugated secondary antibodies (1:500, Molecular probe) for 1 h at room temperature. Cell nuclei were stained with Hoechst 33342 (1 μg/ml, Sigma-Aldrich). After washing with PBS, coverslips were mounted onto slides with Vectashield mounting medium (Vector Laboratories). Images were captured using a lazer scanning confocal microscope (LSM 7000, Carl Zeiss) with x20 objective and processed with ZEN software (Carl Zeiss). The fluorescence intensity of LRRK2 protein in MAP2-positive cells was quantified using Image Pro Plus software (Media Cybernetics). A total of 30–50 neurons in each condition were analyzed.

### Generation of plasmid constructs and transfection

HA-tagged wild-type, full-length human HIF-1α (pcDNA3-HA-HIF-1α-WT) was obtained from Addgene (#18949). The dominant-negative HIF-1α construct (pcDNA3-HA-HIF-1α-DN) was generated by PCR using pcDNA3-HA-HIF-1α-WT as a template. The 1.1-kb product, lacking both the DNA binding and transactivation domains, was cloned into the BamHI and EcoRV sites of pcDNA3-HA vector and confirmed by sequencing. To construct shRNA lentiviral plasmids targeting mouse *Lrrk2* and control shRNAs, two complementary primers were synthesized by Macrogen. As a control, we used a shRNA targeting luciferase, a non-mammalian gene. The primers were annealed and inserted into the Xho I and Hpa I sites of pLL3.7 lentiviral vector (Addgene, #11795) generating pLL3.7-shControl, pLL3.7-shLRRK2 #1, and pLL3.7-shLRRK2 #2. The correct insertion of the shRNA cassette was verified by sequencing. Small interfering RNAs (siRNAs) against mouse HIF-1α and negative control (SN-1002) were synthesized by Bioneer. The siRNA duplexes were as follows: siHIF-1α #1 (forward) 5′-CCCAUUCCUCAUCCGUCAAAU-3′, siHIF-1α #2 (forward) 5′-UGGAUAGCGAUAUGGUCAAUG-3′. All primer sequences used in this study are provided in a Supplementary Table 3. Plasmid or siRNA transfection was performed using Lipofectamine 2000 (Invitrogen), according to manufacturer’s instructions.

### Lenti-virus production

Lentiviral production was performed as previously reported^36^. Briefly, 293FT cells (3–4 × 10^6^ cells) were seeded in a 100-mm culture dish at 24 h before transfection. Then, 4.5 μg of lentiviral construct (pLL3.7-shControl, pLL3.7-shLRRK2 #1, and pLL3.7-shLRRK2 #2), 3 μg of psPAX2 (packaging plasmid, Addgene, #12260), and 1.5 μg of pDM2.G (envoloping plasmid, Addgene, #12259) were co-transfected into 293FT cells using 27 μl of Lipofectamine 2000 (Invitrogen). The medium was changed at 6 h after transfection. Viral supernatants were collected at 48 h after transfection, centrifuged at 500×*g* for 10 min to pellet cell debris. Viral particles were concentrated and purified using a Lenti-X-concentrator (Clontech). Cultured neurons were infected with lentivirus in the presence of 6 μg/ml polybrene (Sigma-Aldrich).

### Luciferase reporter assay

The 2095-bp fragment of mouse *LRRK2* promoter (from −2095 to −1-bp relative to the translation start site) were generated by PCR from genomic DNA of mouse cortical neurons and primers shown in supplementary table 3. The amplified PCR products were subcloned into the TA vector using TOPcloner^TM^ TA core kit (Emzynomics) and confirmed by sequencing (Macrogen). The cloned sequences were released by restriction digestion with KpnI and XhoI and subcloned into the pGL3 basic vector (Promega) at the identical sites. HRE sites in mouse *LRRK2* promoter were mutated by replacing the CG sequences in the HRE consensus sites with AT, using a site-directed mutagenesis kit. To clone the human *LRRK2* promoter reporter construct (pGL3-hLRRK2-Luc), human genomic DNA was isolated from human neuroblastoma SH-SY5Y cells (ATCC) and the 767-bp fragment of human LRRK2 promoter (from −768 to −2 relative to the translation start site) were amplified from genomic DNA by PCR, and then inserted into pGL3 basic vector. Cortical neurons plated in 24-well plates were transfected at DIV8 with the mouse LRRK2 luciferase reporter together with pRL-TK renilla luciferase plasmids. Cells were transfected in serum-free medium by using 0.5 μg of plasmid DNA and 0.5 μl of Lipofectamine 2000 reagent (Invitrogen) per well, according to the manufacturer’s instructions. At two days after transfection, neurons were subjected to scratch injury and luciferase activity was measured at 24 h post-injury using the Dual-Luciferase Reporter Assay system (Promega) according to manufacturer’s instructions. Expression values of firefly luciferase were normalized to respective values of Renilla luciferase.

### Chromatin immunoprecipitation assay

Chromatin immunoprecipitation analysis was performed with the ChIP assay kit (17–295, Millipore), according to manufacturer’s instructions. Briefly, cortical neurons were subjected to scratch injury at DIV 10 and fixed at 24 h post-injury with 1% formaldehyde (w/v in PBS), and sonicated to obtain 100 to 500-bp DNA fragments. Chromatin was immunoprecipitated with 5 μg of anti-HIF-1α (NB100–449, Novus Biologicals) or rabbit IgG (Sigma-Aldrich). The region containing HIF-1α-binding site (5′-CACGC-3′, from −1766 to −1770, relative to the translation start site) on mouse *LRRK2* promoter was amplified by PCR with specific primers. Primer information is provided in Supplementary Table 3.

### Lactate dehydrogenase (LDH) release assay

LDH levels released from damaged cells into the culture medium were measured using CytoTox 96 ^R^ Non-Radioactive Cytotoxicity Assay kit (Promega), according to manufacturer’s instructions. Briefly, at the indicated times after scratch injury or treatment, culture medium was collected and centrifuged at 500×*g* for 5 min to remove the debris. The supernatant was collected and incubated with the LDH reaction mixture at room temperature for 30 min in dark condition. Released LDH level was determined at 490 nm in plate reader (Molecular device).

### TUNEL assay

TUNEL assay was conducted to determine apoptotic cells with the DeadEnd Fluorometric TUNEL system (G3250, Promega) according to manufacturer’s instructions. Briefly, fixed brain sections or cells were permeabilized and incubated with fluorescein TUNEL reaction mixture containing for 60 min at 37°C in dark. The reactions were terminated by immersing the sections in 2 X SSC buffer for 15 min at room temperature. After washing with PBS, nuclei were stained with Hoechst33342 (1 μg/ml in PBS, Sigma-Aldrich). To quantify TUNEL-/DAPI-positive cells, images encompassing 1 mm^2^ of peri-contusional regions or injured neurons were taken using confocal microscope with a 20x objective, and TUNEL-positive cells were counted in cortical layers in three to five coronal sections for each animal,

### Drug treatment

For in vivo experiments, G1023 was dissolved in saline containing 5% dimethyl sulfoxide (DMSO, Sigma-Aldrich) and 4% Tween 80 (Sigma-Aldrich) immediately prior to use. Mice were injected intraperitoneally with either G1023 (50 mg/ml in 0.2 ml volume) or vehicle 3 h before CCI injury. Mice were injected once daily until sacrifice. For in vitro experiments, G1023 (1 μM), GSK2578215A (1 μM), LRRK2-IN-1 (0.1 and 1 μM), MLi-2 (0.3 and 1 μM), and PF-06447475 (0.3 and 1 μM) were dissolved in DMSO and then added to culture media to reach final concentrations at 1 h prior to scratch injury. 2-Methoxyestradiol (2ME2, 0.2 and 1 μM was dissolved in DMSO and then added to culture media to reach final concentrations at 1 h after scratch injury. The final DMSO concentration in the culture medium was 0.05% (v/v).

### Nissl staining and lesion volume measurement

Sections taken between +1.0 mm and −3.5 mm relative to bregma (8–10 sections per brain) were mounted on polysine-coated slides (Thermo Fisher Scientific) and stained for 20 min with 0.5% cresyl violet solution (Sigma-Aldrich). Sections were then dehydrated in graded ethanols (50, 70, 90, and 100%, each for 2 min), cleared in xylene two times for 5 min, cover slipped using Permount mounting medium (Fisher Scientific). All slides were digitally scanned using Aperio ScanScope AT2 slide scanner (Aperio Technologies) with a 20x objective and analyzed using ImageScope software (Aperio Technologies). Lesion areas were assessed in 8 to 10 brain sections per mouse. Lesion volumes were quantified by multiplying the sum of the lesion areas by the distance between sections. Percent lesion volumes were calculated by dividing each lesion volume by the total ipsilateral hemisphere volume (obtained by multiplying the sum of the areas of the ipsilateral hemispheres by the distance between sections). Histological analysis were performed by an investigator who was blinded to the experimental groups.

### Beam balance test

Beam balance test was assessed using the previous method^32^ with some modifications. Briefly, mice were placed on the middle of a wooden rounded bar (5 mm diameter, 90 cm long, 50 cm height) and scored as follows: mice unable to stay on the beam for 30 s, 0 points; mice unable to move but able to stay on the beam for 30 s, 1 point; mice that attempt to turn to the right or left side of the beam without walking, 2 points; mice turning to right or left side and walk on the beam with more than one step, 3 points; mice able to traverse the beam with more than 50% of foot slip of the affected hind limb, 4 points; mice able to transverse the bean with less than 50% foot slip of the affected hind limb, 5 points; mice able to transverse the beam with not more than one foot slip, 6 points. Behavior tests were carried out by an investigator who was blinded to the experimental groups.

### Novel object recognition (NOR) test

NOR test was performed at 7 day after CCI, according to a previous method^32^ with some modifications. Mice were placed in a black, wooden, no-top square box (45 × 45 cm size, 25-cm-high walls) and allowed to explore two objects for 30 min (habituation and familiarization). After 4 h, mice were returned to the box with the familiar objects for 5 min in the box (Training). And then mice were placed back in the box with one new object and one familiar object for 5 min at 1 h post-training (Testing). Time spent exploring the new or the familiar object was measured during the last 5 min of exploration (test) and Discrimination index (DI) was calculated as follows: DI = (time spent exploring the new object−time spent exploring the familiar object)/(total time spent exploring both objects). Behavior tests were carried out by an investigator who was blinded to the experimental groups.

### Statistical analysis

All of the experiments were randomized and performed in a blinded manner. All data were either averages or representative data from at least three independent experiments. The number of independent experiments and group size are indicated in the figure legends. Statistical analyses were conducted using the software Graph-Pad Prism (GraphPad Software 5, Inc.). Prior to determining statistical significance, Shapiro-Wilk test was performed to assess normality. We used appropriate statistical tests, based on the comparison and population. For two unpaired comparison, two-tailed Student *t* test or Mann–Whitney *U*-test was performed. For multiple comparison, one-way ANOVA followed by appropriate post hoc test (Dunnett or Newman-Keuls post hoc test) was performed. Animal behavior data were analyzed by two-way ANOVA with Bonferroni post hoc test. Data were expressed as mean ± s.d. or s.e.m. *, **, *** and **** in the figures denote *p* < 0.05, 0.01, 0.001,  and 0.0001, respectively.

## Results

### Brain injury induces LRRK2 expression in neurons

**Fig. 1:**
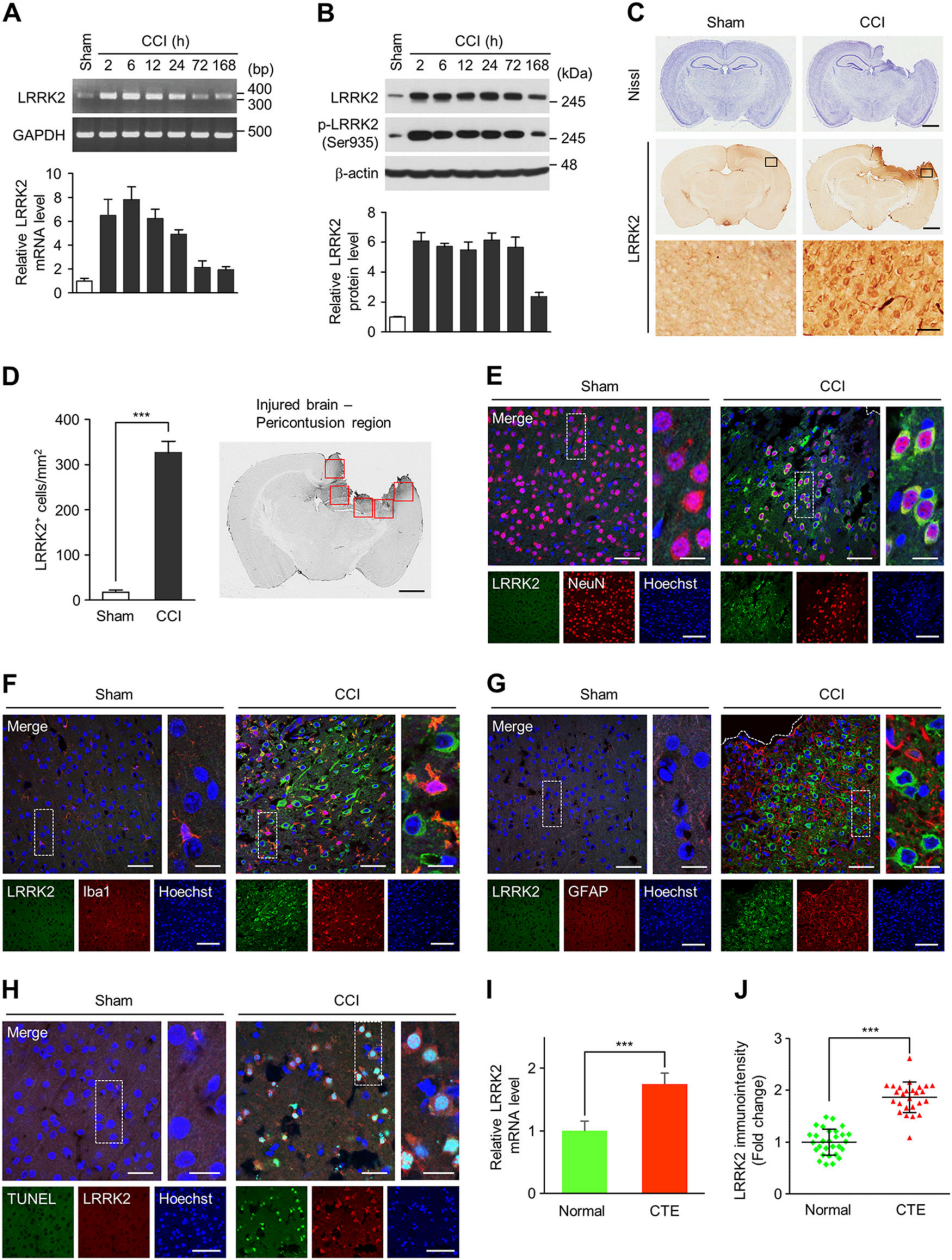
LRRK2 induction in in vivo TBI models. Levels of *LRRK2* mRNA (**a**) and total and phospho-S935 (pS935) LRRK2 protein (**b**) in brain lysates from the ipsilateral side of sham and CCI group. Shown are representative gel images (**a**, top) or blots (**b**, top) and quantification of *LRRK2* mRNA (**a**, bottom) and LRRK2 and pS935 LRRK2 protein levels (**b**, bottom) relative to sham. Bar graph shows means ± s.d. (*n* = 3). **c** Nissl staining (upper row) and LRRK2 immunostaining (middle and lower rows) from coronal brain sections of sham and CCI group at 24 h post-injury. Scale bar = 1 mm (upper and middle rows) and 50 μm (lower row). **d** Numbers of LRRK2-positive cells (per mm^2^) in pericontusion area of CCI and corresponding area of sham group. Bar graph shows mean ± s.d. (*n* = 3). Pericontusion areas in the injured brain used for quantification of LRRK2 are shown in right. Scale bar = 1 mm. **e–g** Sham (left) and CCI-injured (right, at 24 h post-injury) brain sections immunostained with LRRK2 and NeuN (**e**), Iba1 (**f**), or GFAP (**g**) antibodies. Scale bar = 50 μm (left merge), 5  μm (right merge), and 100 μm (lower row). **h** TUNEL staining and LRRK2 immunostaining in sham (left) and CCI-injured (right, at 24 h post-injury) brain sections. Scale bar = 25  μm (left merge), 5 μm (right merge), and 50 μm (lower low). **i** Level of *LRRK2* mRNA was analyzed by quantitative real-time PCR from postmortem brains of normal subjects (*n* = 7) and CTE patients (*n* = 7). Quantification of *LRRK2* mRNA level relative to normal is presented as means ± s.d. **j** Densitometry analysis of LRRK2 immunostaining in neurons (*n* = 29) of CTE postmortem brain and neurons (*n* = 26) of normal brain. Means ± s.d. Student *t* test was performed for all experiments. ***p < 0.001

To investigate the involvement of LRRK2 in injury, we exploited the CCI injury, a widely used model to study the mechanism of TBI and to evaluate therapies^37^. In the peri-contusional brain region of the CCI group, we observed a rapid and robust induction of *LRRK2* mRNA within 2 h post-injury, which sustained for about a day, followed by a gradual decrease (Fig. 1a). LRRK2 protein level also increased within 2 h post-injury, which was sustained for 3 days (Fig. 1b). LRRK2 immunoreactivity (Fig. 1c) and the number of LRRK2-positive cells (Fig. 1d) was significantly increased in peri-contusional brain regions in the cortex and the hippocampus of the CCI group but not in the contralateral side of the CCI group or the sham-operated control group. Using additional antibodies against LRRK2 (Supplementary table 2), we further verified the induction of LRRK2 after CCI injury (Supplementary Fig. 1). The specificity of immunoreactivity was confirmed by using two control IgG antibodies (Supplementary Fig. 2).

LRRK2 induction by CCI injury was primarily detected in NeuN-positive neurons (Fig. 1e), which was evident within 2 h post-injury and sustained for 3 days (Supplementary Fig. 3). Iba1-positive microglia also increased LRRK2 level but at a later time point and to a much lesser extent (Fig. 1f and Supplementary Fig. 4). No significant increase was detected in GFAP-positive astrocytes up to 7 days (Fig. 1g and Supplementary Fig. 5). LRRK2 expression in neurons triggered cell death, as evidenced by the increase in the number of TUNEL and LRRK2 double-positive cells (Fig. 1h and Supplementary Fig. 6).

Next, we examined the levels of LRRK2 mRNA and protein in postmortem brains from patients with CTE, a neurodegenerative disease found in people who had multiple head injuries. Consistent with the rodent CCI injury model, we detected a significant increase in *LRRK2* mRNA level (Fig. 1i) and LRRK2 immunoreactivity (Fig. 1j and Supplementary Fig. 7) in CTE postmortem brains compared to control brains.

### LRRK2 expression is induced by several in vitro models of injury

**Fig. 2:**
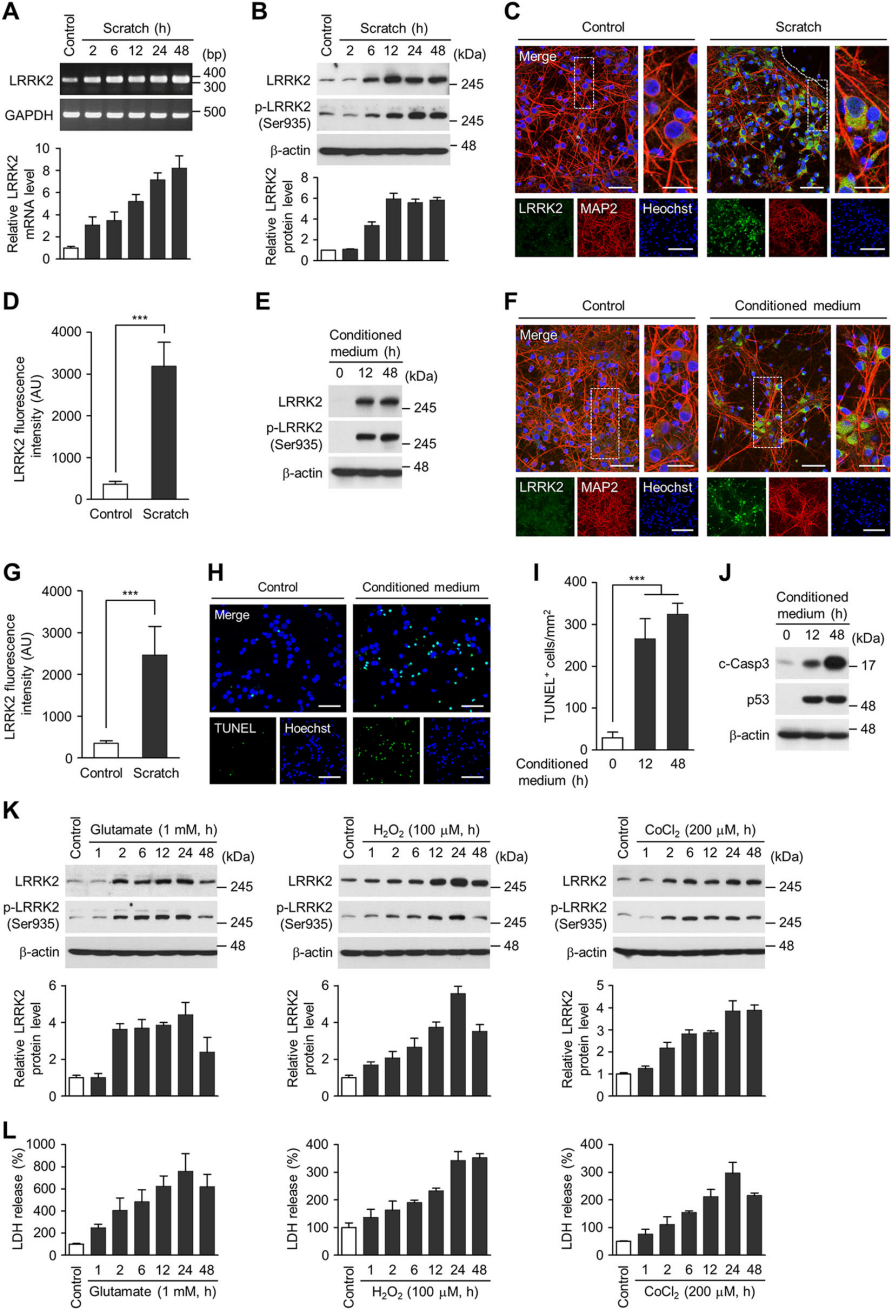
LRRK2 induction in in vitro TBI models. Levels of *LRRK2* mRNA (**a**) and total and phospho-S935 (pS935) LRRK2 protein levels (**b**) in control and scratch-injured neurons. Shown are representative gel images (**a**, top) or blots (**b**, top) and quantification of *LRRK2* mRNA (**a**, bottom) and LRRK2 and pS935 protein levels (**b**, bottom) relative to control. Bar graph shows means ± s.d. (*n* = 3). **c** Representative images of control (left) and scratch-injured (right, at 48 h post-injury) cortical neurons immunostained with LRRK2 and MAP2 antibodies. Scale bar = 50 μm (left merge), 10 μm (right merge), and 25 μm (lower low). **d** Quantification of LRRK2 fluorescence intensity in MAP2-positive cells. Bar graph shows mean ± s.d. (*n* = 3). **e–j** Conditioned medium was collected from scratch-injured cortical neurons at 6 h post-injury and applied to intact cortical neurons at DIV 10. Intact cortical neurons treated with the conditioned medium were subjected to western blot analysis (**e**, **j**) or immunocytochemistry (**f**, **g**). **e** Levels of total and pS935 LRRK2 in cortical neurons after treatment with conditioned medium for 0, 12, and 48 h. **f** Control (left) and conditioned medium-treated (right) cortical neurons immunostained with LRRK2 and MAP2 antibodies. Scale bar = 50 μm (left merge), 10 μm (right merge), and 20 μm (lower low). **g** Quantification of LRRK2 fluorescence intensity in MAP2-positive cells. Bar graph shows mean ± s.d. (*n* = 3). **b** TUNEL staining in control (left) and conditioned medium-treated (right) cortical neurons. **i** Numbers of TUNEL-positive cells (per mm^2^) after treatment with conditioned medium for 0, 12, and 48 h. Bar graph shows mean ± s.d. (*n* = 3). **j** Levels of cleaved caspase-3 and p53 after treatment with conditioned medium for 0, 12, and 48 h. **k** Levels of total and pS935 LRRK2 in cortical neurons treated with 1 mM glutamate, 100 μM H_2_O_2_, and 200 μM CoCl_2_. Shown are representative immunoblots (top) and quantification of LRRK2 protein level (bottom). (**l**) LDH release assay. Bar graph shows mean ± s.d. (*n* = 3). Student *t* test was performed for (**d**) and (**g**) and One-way ANOVA followed by Dunnett’s post hoc test was performed for (**i**). ***p < 0.001. Images presented are representative of at least three independent experiments

Scratch injury recapitulates certain aspects of TBI in that the injury is initiated by a physical, primary damage, followed by a secondary deterioration mediated by detrimental factors released from damaged cells. Consistent with the results in vivo (see Fig. 1), we detected a marked increase in LRRK2 mRNA and protein levels after scratch injury in primary cortical neurons (Fig. 2a–d). The conditioned medium collected from scratch-injured primary cortical neurons was sufficient to induce LRRK2 expression when added to intact neuronal culture (Fig. 2e–g), suggesting that soluble factors secreted by damaged cells played a role. The conditioned medium also increased the number of TUNEL-positive cells (Fig. 2h, i) and induced caspase-3 activation and the expression of p53 (Fig. 2j), indicative of neurotoxicity.

We also detected a robust induction of LRRK2 expression in response to glutamate, hydrogen peroxide, and cobalt chloride (CoCl_2_) (Fig. 2k). Cytotoxicity of such stimuli was confirmed by LDH release (Fig. 2l). These results suggest that the expression of endogenous LRRK2 is regulated by multiple pathogenic assaults.

### LRRK2 expression is transcriptionally regulated by HIF-1α

**Fig. 3:**
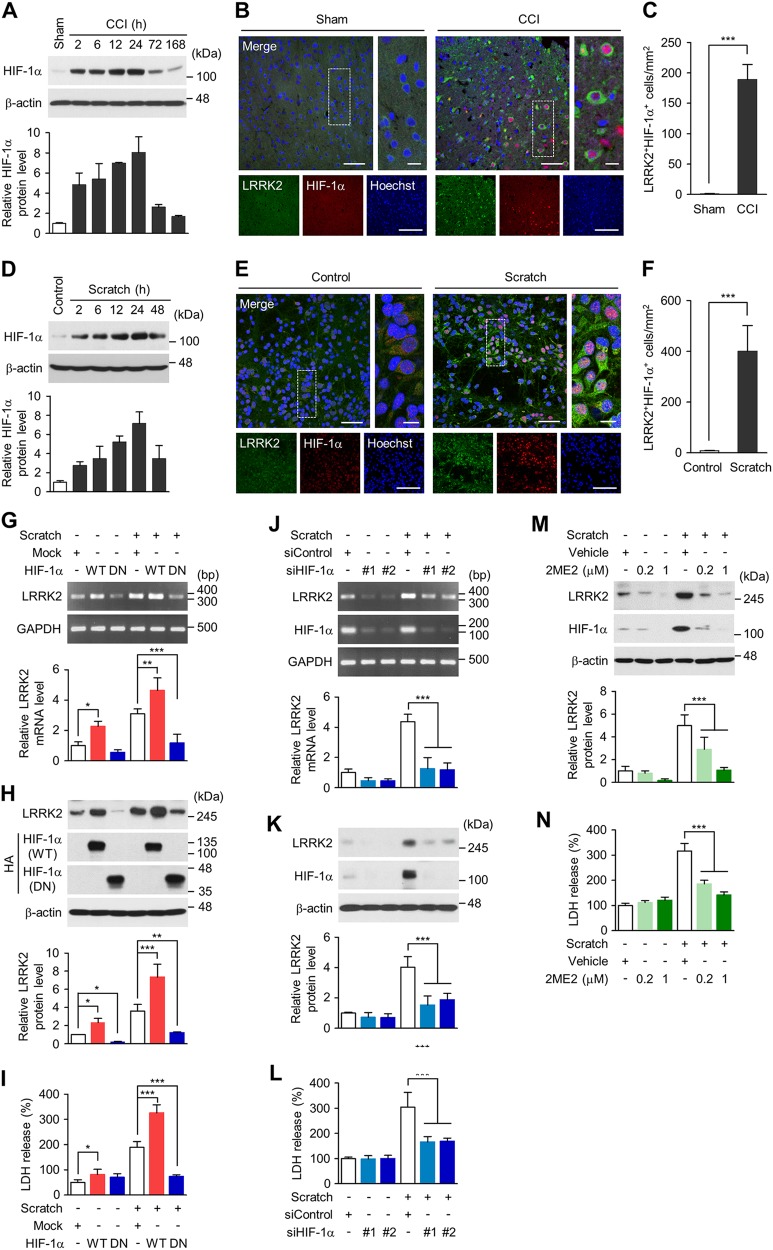
HIF-1α dependent upregulation of LRRK2. **a** Level of HIF-1α protein in brain lysates from the ipsilateral side of sham and CCI group. Shown are representative blots (top) and quantification of HIF-1α protein level (bottom) relative to sham. Bar graph shows means±s.d. (*n* = 3). **b** Representative images of sham (left) and CCI-injured (right, at 48 h post-injury) brain sections immunostained with LRRK2 and HIF-1α antibodies. Scale bar = 50 μm (left merge), 5 μm (right merge), and 25 μm (lower low). **c** Numbers of LRRK2 and HIF-1α double-positive cell (per mm^2^) in sham and CCI-injured brain sections. Bar graph shows mean ± s.d. (*n* = 3). **d** Level of HIF-1α protein in control and scratch-injured cortical neurons. Representative immunoblots (top) and quantification of HIF-1α level relative to control (bottom) are shown. Bar graph shows means ± s.d. (*n* = 3). **e** Representative images of control (left) and scratch-injured (right, at 24 h post-injury) cortical neurons immunostained with LRRK2 and HIF-1α antibodies. Scale bar = 50  μm (left merge), 5 μm (right merge), and 25 μm (lower low). **f** Numbers of LRRK2 and HIF-1α double-positive cells (per mm^2^) in control and scratch-injured cortical neurons. Mean  ± s.d. (*n* = 3). **g**–**n** Cortical neurons were transfected with wild-type (WT) or dominant-negative (DN) of HA-tagged HIF-1α or with two different siRNA against HIF-1α at DIV 8, and cortical neurons were scratched at DIV 10. Levels of *LRRK2* mRNA (**g**, **j**) and LRRK2 protein (**h**, **k**) were examined at 24 h post-injury and normalized to those of mock (**g**, **h**) or control siRNA-transfected neurons (**j**, **k**). Bar graph shows means ± s.d. (*n* = 3). **i**, **l** LDH release assay. Bar graph shows mean ± s.d. (*n* = 3). **m**, **n** 2ME2 (0.2 and 1 μM) or vehicle (0.05% DMSO) was treated at 1 h post-injury. **m** Levels of LRRK2 and HIF-1α in cortical neurons treated with 2ME2. Bar graph shows means ± s.d. (*n* = 3).**n** LDH release assay. Bar graph shows means ± s.d. (*n* = 3). Student *t* test was performed for **c** and **f** and One-way ANOVA followed by Newman–Keuls post hoc test was performed for **g** to **n**. *p < 0.01; **p < 0.005; ***p < 0.001

Cobalt chloride is a chemical inducer of HIF-1α, and glutamate and hydrogen peroxide are known to stabilize or increase the level of HIF-1α^38,39^. In the promoter region of mouse *LRRK2* gene, we found multiple hypoxia-response elements (HREs), putative binding sites for HIF-1, suggesting that HIF-1α might play a role in the induction of LRRK2. CCI injury induced a robust increase in the protein level of HIF-1α (Fig. 3a–c), and LRRK2 was detected in HIF-1α-positive cells (Figs. 3b, c). Scratch injury also increased the protein level of HIF-1α in cortical neurons (Fig. 3d) and the number of HIF-1α and LRRK2 double-positive cells (Fig. 3e, f). The level of *HIF-1α* mRNA, however, remained unaltered in both CCI and scratch injury models (Supplementary Fig. 8).

Conditioned media collected from scratch-injured primary cortical neurons again was sufficient to increase the protein level of HIF-1α when added to intact cortical neuronal culture (Supplementary Fig. 9A), reminiscent of LRRK2 mRNA induction (see Fig. 2e–g). The protein level of HIF-1α increased by other assaults that induced LRRK2 expression, such as glutamate, H_2_O_2_, and CoCl_2_ (Supplementary Figs. 9B–D).

We then examined if the mRNA and protein levels of LRRK2 were affected by regulating the expression or stability of HIF-1α. Overexpression of HA-tagged, wild-type (WT) HIF-1α (HA-HIF-1α-WT) in primary cortical neurons increased the basal and the scratch injury-induced elevation of LRRK2 mRNA (Fig. 3g) and protein levels (Fig. 3h). Conversely, overexpression of dominant-negative (DN) HIF-1α (HA-HIF-1α-DN), which lacks the DNA-binding domain, substantially decreased the basal and the scratch injury-induced up-regulation of LRRK2 mRNA (Fig. 3g) and protein levels (Fig. 3h). Scratch injury-induced increases in LRRK2 mRNA and protein levels were nearly completed blocked by two different small interfering RNAs (siRNAs) against HIF-1α (siHIF-1α) (Fig. 3j, k) or treatment with 2-methoxyestradiol (2ME2) (Fig. 3m), which down-regulates HIF-1α at the posttranscriptional level^40^. Manipulation of the level or activity of HIF-1α by HIF-1α-WT or HIF-1α-DN, siHIF-1α, or 2ME2 increased or decreased cytotoxicity in a way which was consistent with the changes in the level of LRRK2 (Fig. 3i–n). These results demonstrate that HIF-1α regulates LRRK2 expression and that activation of the HIF-1α-LRRK2 axis has detrimental effects on neurotoxicity.

**Fig. 4:**
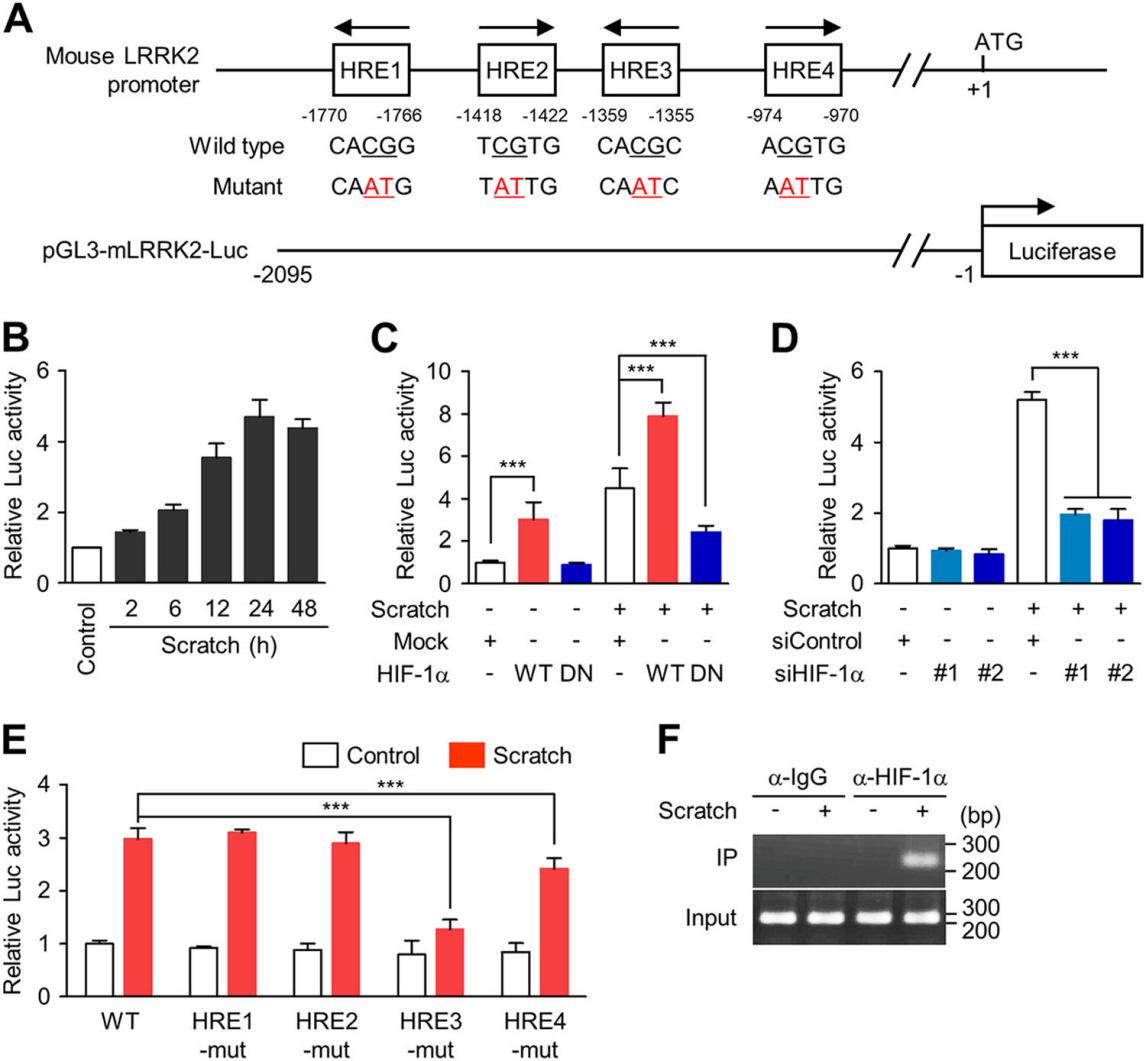
Transcriptional regulation of LRRK2 by HIF-1α. **a** Schematic linear maps of mouse *LRRK2* promoter, indicating location and nucleotide sequence of four putative HRE sites and mutagenesis information of each HRE sites. **b** At DIV 8, pGL3-mLRRK2-Luc was transfected into primary cortical neurons, followed by scratch injury at DIV 10, and luciferase activity was measured at 48 h post-injury. Bar graph shows means ± s.d. (*n* = 3). **c**, **d** Primary cortical neurons were co-transfected with pGL3-mLRRK2-Luc and wild-type (WT) or dominant-negative (DN) of HIF-1α (**c**) or with two different siRNA against HIF-1α (**d**) at DIV 8. Neurons were scratched at DIV 10, and luciferase assay was performed with cell lysates at 48 h post-injury. Bar graph shows means ± s.d. (*n* = 4). **e** Primary cortical neurons were transfected with pGL3-mLRRK2-Luc or HRE mutant constructs which harbor mutations in one of the four HRE sites. Bar graph shows means ± s.d. (*n* = 3). **f** Control and scratch-injured cortical neurons were processed for chromatin immunoprecipitation (ChIP) with anti-HIF-1α or IgG control antibodies. One-way ANOVA followed by Newman–Keuls post hoc test was performed for all experiments. ***p < 0.001

Using fuzznuc-EMBOSS software (http://emboss.bioinformatics.nl/cgi-bin/emboss/fuzznuc), we analyzed the proximal promoter region of mouse *LRRK2* gene and identified four putative HRE sites, including two HREs oriented on the sense strand and two on the antisense strand (Fig. 4a). To determine whether the HRE sites were transcriptionally active, we constructed a mouse LRRK2 reporter plasmid, pGL3-mLRRK2-Luc and performed a reporter assay in primary cortical neurons. Scratch injury significantly increased the luciferase activity of pGL3-mLRRK2-Luc compared to pGL3 basic control (Fig. 4b). In the human *LRRK2* promoter, seven putative HRE sites were identified (Supplementary Fig. 10A), and we confirmed that pcDNA3-HA-HIF-1α-WT markedly increased the luciferase activity of the human LRRK2 reporter plasmid, pGL3-hLRRK2-Luc in human neuroblastoma SH-SY5Y cells (Supplementary Fig. 10B), suggesting that transactivation of *LRRK2* by HIF-1α may be a conserved mechanism.

To confirm HIF-1α-dependent transactivation of *LRRK2*, either pcDNA3-HA-HIF-1α-WT or pcDNA3-HA-HIF-1α-DN were co-transfected with pGL3-mLRRK2-Luc or a pGL3-basic control plasmid. Both the basal and the scratch injury-induced elevation of luciferase activity were markedly increased by HIF-1α-WT (Fig. 4c) but substantially prevented by HIF-1α-DN (Fig. 4c), and transfection with two different siHIF-1α prevented the scratch-injury induced increase of luciferase activity (Fig. 4d). Next, we generated mutants for each of the four putative HREs (Fig. 4a) and found that HRE3-mut nearly completely prevented the increased in luciferase activity induced by scratch injury (Fig. 4e). HRE1-mut and HRE2mut had little effect and HRE4-mut slightly reduced luciferase activity. Furthermore, by chromatin immunoprecipitation assay, we demonstrated scratch injury-inducible, direct binding of HIF-1α to HRE3 (Fig. 4f). Together, these results suggest that HIF-1α induces the expression of LRRK2 by binding directly to the HRE site, primarily HRE3, in *LRRK2* proximal promoter.

### LRRK2 causes neuronal toxicity after scratch injury

**Fig. 5:**
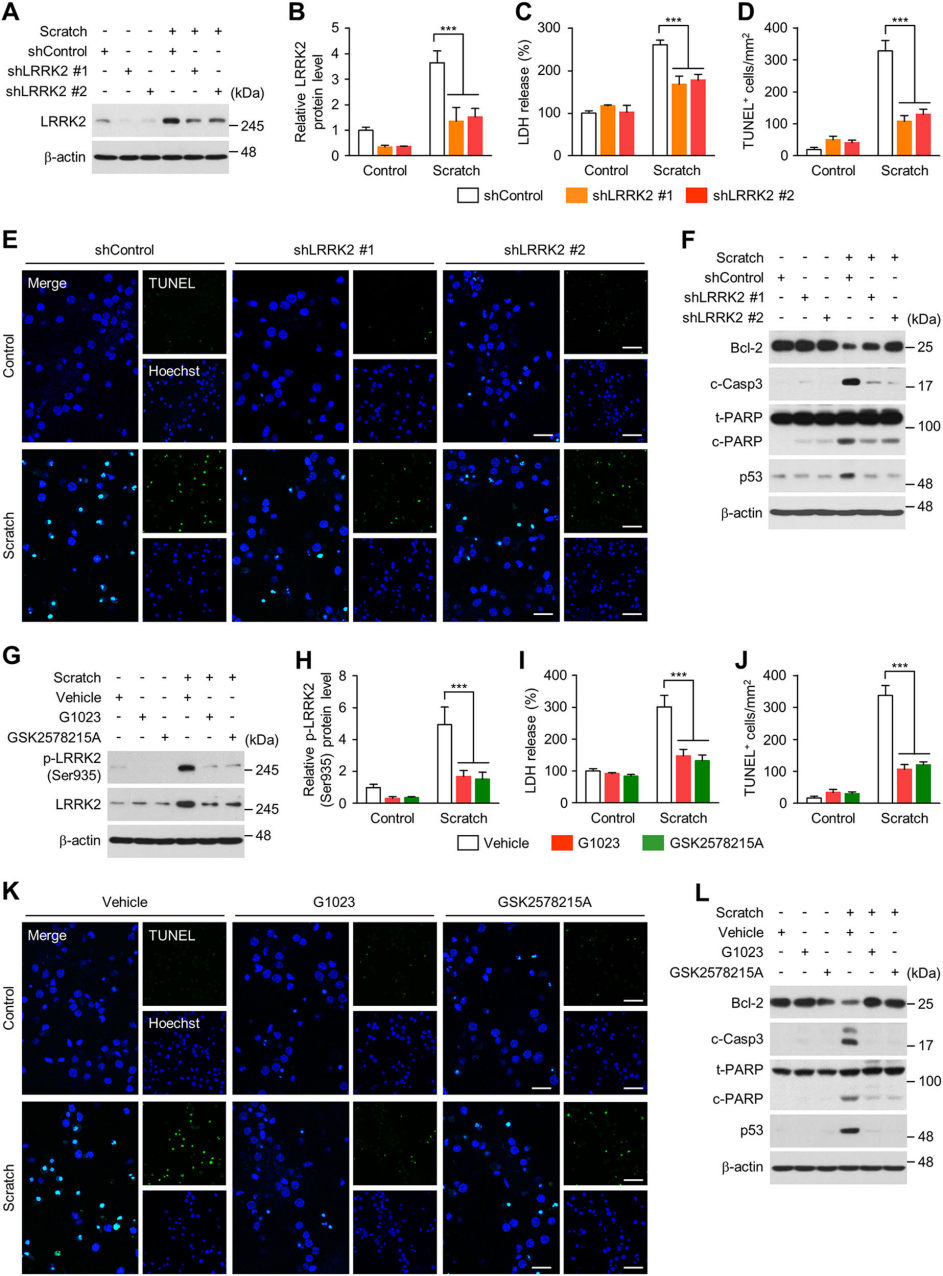
LRRK2-mediated neuronal cell death after scratch injury. **a-f** Lentivirus (pLL3.7-shControl, shLRRK2 #1 or #2) was infected into cortical neurons at DIV 8, followed by scratch injury at DIV 10. After 48 h, the analyses described below were performed. **a**, **b** Levels of LRRK2 protein in control and scratch-injured cortical neurons infected with shControl or shLRRK2-lentivirus. Representative immunoblots (**a**) and quantification (**b**) of LRRK2 protein levels are shown. Bars in **b** show means ± s.d. (*n* = 3). **c** LDH release assay. LDH release level relative to control neurons infected with shControl-lentivirus. Bar graph shows means ± s.d. (*n* = 3). **d**, **e** TUNEL assay. Numbers of TUNEL-positive cells (per mm^2^) in control and scratch-injured cortical neurons infected with shControl or shLRRK2-lentivirus (**d**) and representative images (**e**) are presented. Bar graph in **d** shows means ± s.d. (*n* = 3). Scale bar in **e** = 50 μm (merge) and 100 μm (right column). **f** Immunoblots of control and scratch-injured cortical neurons infected with shLRRK2 or shControl, using antibodies against Bcl-2, cleaved caspase-3, total and cleaved PARP, and p53. β-actin antibodies were used as a loading control. **g-l** Cortical neurons were pretreated with LRRK2 kinase inhibitors (1 μM G1023 or 1 μM GSK2578215A) for 1 h prior to scratch injury at DIV 10. After 48 h, the analyses described below were performed. **g**, **h** Levels of total and phospho-S935 LRRK2 protein in control and scratch-injured cortical neurons treated with G1023, GSK2578215A, or vehicle control. Representative immunoblots (**g**) and quantification (**h**) of phospho-LRRK2 are shown. Bars in (**h**) show means ± s.d. (*n* = 3). **i** LDH release assay. LDH release level relative to control neurons treated with vehicle control. Bar graph shows means ± s.d. (*n* = 3). **j**, **k** TUNEL assay. Numbers of TUNEL-positive cells (per mm^2^) in control and scratch-injured cortical neurons treated with LRRK2 inhibitors or vehicle control (**d**) and representative images (**k**) are presented. Bar graph in J shows means ± s.d. (*n* = 3). Scale bar in **k** = 50 μm (merge) and 100 μm (right column). **l** Immunoblots of control and scratch-injured cortical neurons treated with LRRK2 inhibitors or vehicle control, using antibodies against Bcl-2, cleaved caspase-3, total and cleaved PARP, and p53. β-actin antibodies were used as a loading control. One-way ANOVA followed by Newman-Keuls post hoc test was performed for all experiments. ***p < 0.001

To evaluate the functional consequence of LRRK2 induction after scratch injury, we first generated lentiviral shRNA targeting LRRK2 (pLL3.7-shLRRK2 #1 and #2) and confirmed that LRRK2 was efficiently down-regulated in primary cortical neurons (Fig. 5a, b). LDH release assay and TUNEL assay revealed that lenti-shLRRK2 substantially attenuated scratch injury-induced cytotoxicity (Fig. 5c–e). Up-regulation of cell death markers, such as cleaved caspase-3, cleaved PARP, and p53, induced by scratch injury was also substantially reduced (Fig. 5f). Using multiple inhibitors of LRRK2, G1023, GSK2578215A, LRRK2 IN-1, PF-06447475, and MLi-2 (Fig. 5g, h and Supplementary Fig. 11), we found that pharmacological inhibition of LRRK2 prevented the scratch injury-induced neuronal toxicity (Fig. 5i–k and Supplementary Fig. 11) as well as the increase of cell death markers (Fig. 5l).

To investigate the signaling pathways involved in neuronal cell death downstream of the induction and activation of LRRK2, we examined the involvement of the apoptosis signal-regulating kinase (ASK1)-mitogen-activated protein kinase kinase (MKK)-mitogen-activated protein kinase (MAPK) cascade. ASK1 is a member of MAP3K and activates c-Jun N-terminal kinase (JNK), which induces degeneration of dopaminergic neurons in LRRK2 transgenic mouse^41^. Scratch injury induced phosphorylations of ASK1, MKK4, JNK, and c-Jun, which were substantially prevented by transfection of lenti-shLRRK2 (Supplementary Fig. 12A) or treatment with kinase inhibitors of LRRK2 (Supplementary Fig. 12B). These results suggest that injury-induced induction of LRRK2 causes neuronal toxicity, perhaps by activating the ASK1-MKK4-JNK/c-Jun pathway.

### LRRK2 kinase inhibitor reduces CCI-induced brain lesion and cell death

**Fig. 6:**
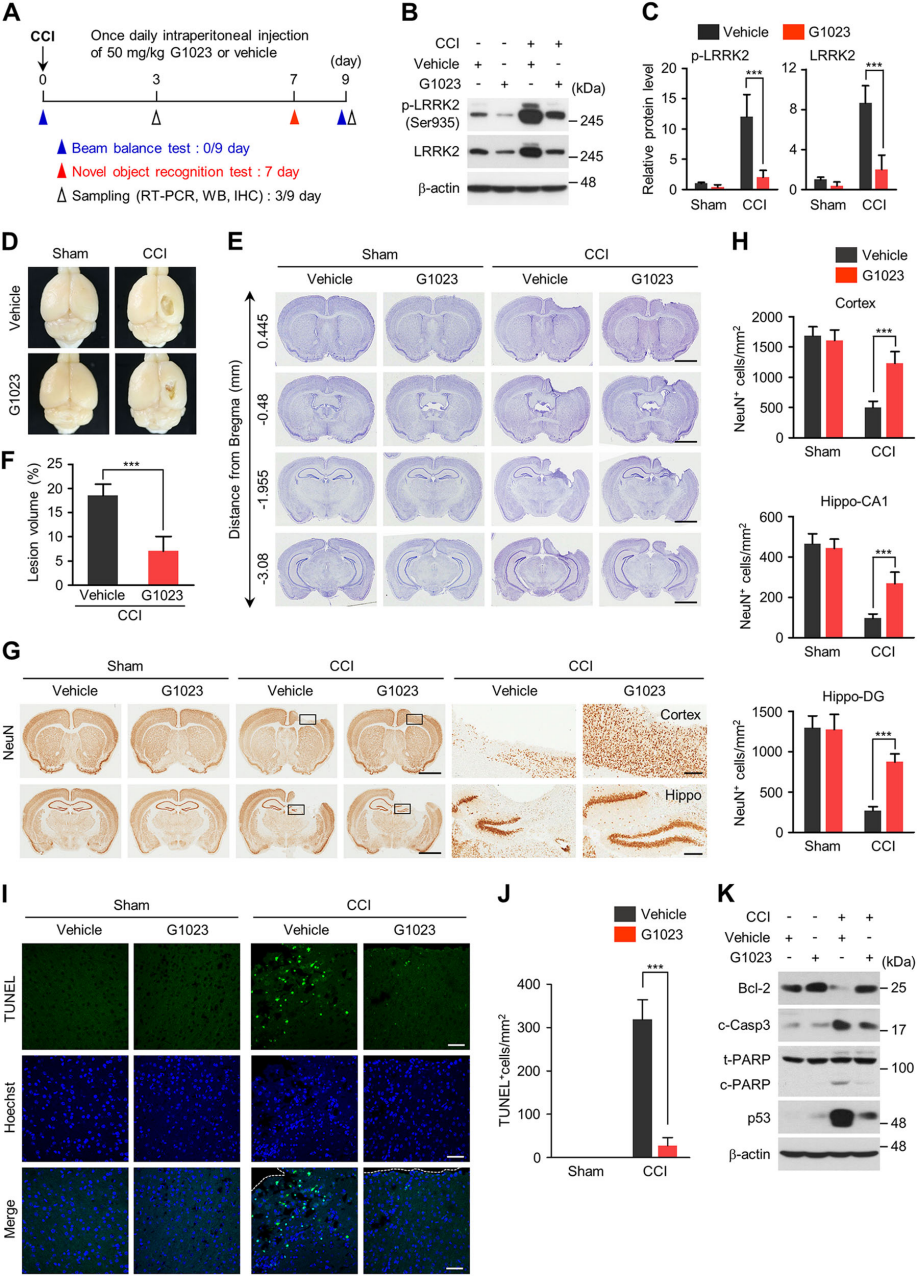
LRRK2 inhibition alleviates CCI-induced brain lesions and neuronal loss. **a** Experiment scheme for drug application, behavioral tests, and brain preparation. **b**, **c** Levels of total and phospho-S935 LRRK2 in brain lysates from each experiment group. β-actin antibodies were used as a loading control. Representative immunoblots (**c**) and quantification of total and phospho-LRRK2 levels relative to vehicle-injected sham group are presented. Bar graph in C shows means ± s.d. (*n* = 4–5). **d** Representative whole brain images of sham and CCI group. **e** Nissl staining of coronal brain sections from sham and CCI group. Scale bar = 2 mm. **f** Quantification of lesion volume, which is presented as percentage of total ipsilateral hemisphere. Bar graph shows means ± s.d. (*n* = 4–5). **g** Coronal brain sections from sham and CCI group treated with G1023 or vehicle control immunostained with NeuN antibodies. Magnified images of cortical and hippocampal regions are presented in right. Scale bar = 2 mm (whole brain sections) and 50 μm (magnified images). **h** Numbers of NeuN-positive cells (per mm^2^) in brain sections from sham and CCI groups treated with G1023 or vehicle control. Bar graphs show means ± s.d. (*n* = 4–5). **i**, **j** TUNEL assay. Representative image (**i**) and numbers (**j**) of TUNEL-positive cells (per mm^2^) in sham and CCI groups treated with G1023 or vehicle control are presented. Bar graph in (**j**) shows means ± s.d. (*n* = 4–5). Scale bar in **i** = 50 μm. **k** Immunoblots of sham and CCI groups treated with G1023 or vehicle control, using antibodies against Bcl-2, cleaved caspase-3, total and cleaved PARP, and p53. β-actin antibodies were used as a loading control. One-way ANOVA followed by Newman–Keuls post hoc test was performed for (**c**), (**h**), and (**j**) and Student *t* test was performed for (**f**). ***p < 0.001

We next examined whether inhibition of LRRK2 kinase activity could alleviate the neurologic deficits after CCI injury. For this purpose, G1023, a brain permeable, kinase inhibitor of LRRK2^42^ was administrated three hour prior to CCI injury and once daily for 9 days after CCI injury by intraperitoneal injection (Fig. 6a). Administration of G1023 substantially prevented the CCI injury-induced increase of total and pS935 LRRK2 (Figs. 6b, c). We found that CCI injury-induced brain lesion volume (Fig. 6d) was markedly reduced by G1023 (Fig. 6d–f). Notably, CCI-induced neuronal loss in the cortex and the hippocampus (CA1 and dendate gyrus subregions) was significantly suppressed by G1023 (Fig. 6g, h). Protective effect of G1023 was also confirmed in the TUNEL assay (Fig. 6i, j). In the CCI-injured brain, G1023 prevented both the increase of pro-apoptotic proteins, such as cleaved caspase-3, cleaved PARP1, and p53 and the decrease of anti-apoptotic protein, Bcl-2 (Fig. 6k). In addition, G1023 blocked the activation of ASK1-MKK4-JNK/c-Jun cascade induced by CCI (Supplementary Fig. 13). Together, these results show that administration of G1023 reduces brain lesion and protects CCI-injured brain from neurotoxicity.

### LRRK2 kinase inhibitor improves neuroinflammation, brain edema, and blood-brain barrier (BBB) breakage

**Fig. 7:**
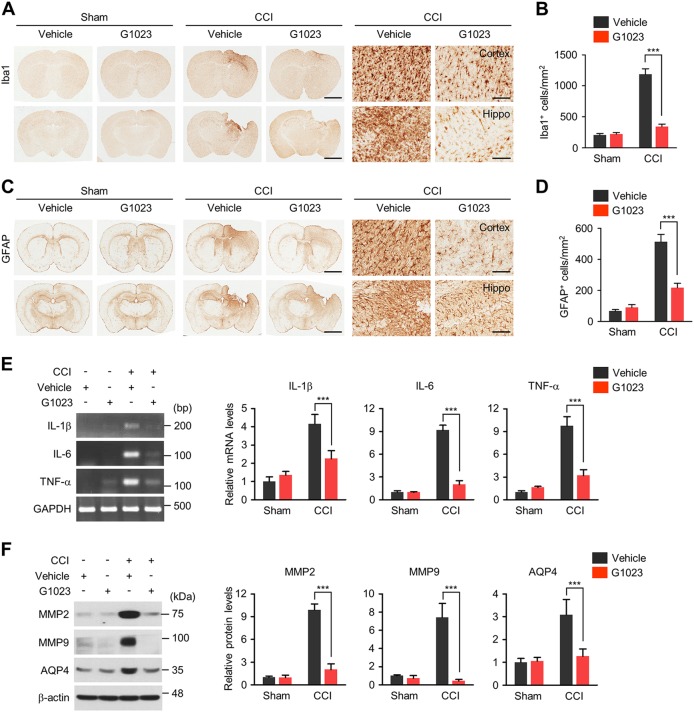
LRRK2 inhibition reduces CCI-induced brain pathologies. **a**, **c** Representative images of brain sections from sham or CCI groups treated with G1023 or vehicle control, using antibodies against Iba1 (**a**) or GFAP (**c**). Scale bar = 2 mm. Magnified images of cortical and hippocampal regions are presented in right. Scale bar = 50 μm. Numbers of Iba1 (**b**) or GFAP (**d**) positive cells (per mm^2^) in each experiment group are presented. Bar graphs in (**b**) and (**d**) show means ± s.d. (*n* = 4–5). **e** Levels of proinflammatory cytokine mRNAs (*IL-1β*, *IL-6*, and *TNF-α*) in each experiment group was analyzed by RT-PCR. Quantification of mRNA level relative to vehicle-injected sham control is presented as mean ± s.d. (*n* = 4–5). **f** Levels of MMP2, MMP9 and AQP4 proteins in each experiment group. Quantification of each protein level relative to vehicle-injected sham group is presented as mean ± s.d. (*n* = 4–5). One-way ANOVA followed by Newman–Keuls post hoc test was performed for all experiments. ***p < 0.001

Neuroinflammation, edma, and BBB breakage are associated with both acute neurological dysfunctions and long-lasting disabilities in TBI patients. CCI injury markedly increased the numbers of Iba1^+^ (Fig. 7a, b) or GFAP^+^ (Fig. 7c, d) cells in the peri-contusional regions of the ipsilateral cortex and the hippocampus, and both numbers were reduced by G1023. In addition, a robust induction of pro-inflammatory cytokines, such as *IL-1β*, *IL-6*, and *TNF-α* mRNA in response to CCI injury was markedly prevented by G1023 (Fig. 7e). We also monitored if LRRK2 inhibition could protect the brain from BBB breakage and edema by measuring the levels of matrix metalloproteinase (MMP) 2 and MMP9 as markers of BBB breakage and aquaporin (AQP) 4 as a marker of edema (Fig. 7f). Upregulation of MMP2, MMP9, and AQP4 induced by CCI was nearly completely suppressed by administration of G1023.

### LRRK2 kinase inhibitor ameliorates CCI-induced motor and cognitive deficits

**Fig. 8:**
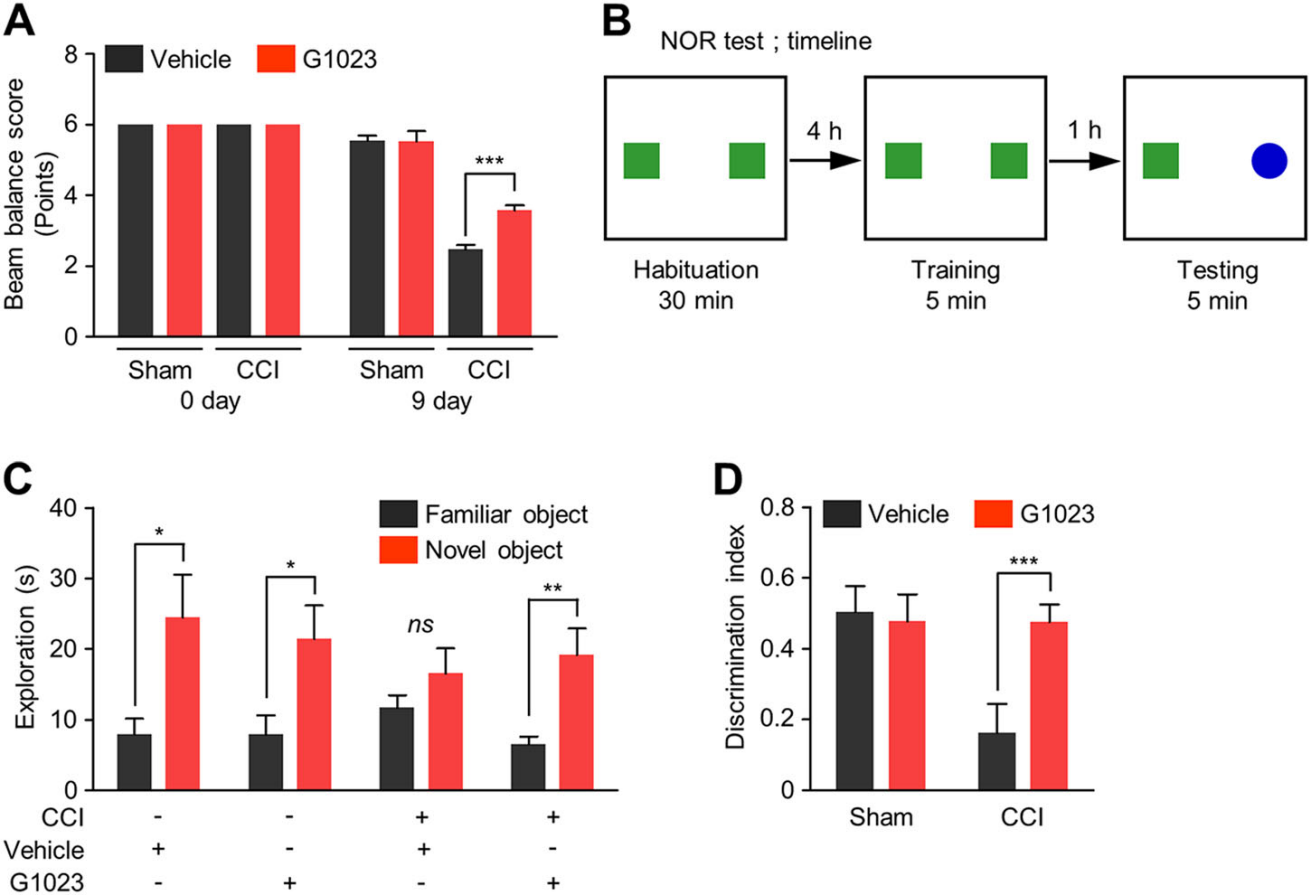
LRRK2 inhibition ameliorates motor and cognitive deficits induced by CCI injury. Beam balance test and novel objective recognition (NOR) test were performed at 7 and 9 days post-injury, respectively. **a** Beam balance score in each experimental group. Means ± s.e.m. (*n* = 8). **b** Schematic diagram of NOR test. **c** Exploration time for a familiar and a novel object and (**d**) discrimination index in NOR test in each experimental group. Means ± s.e.m. (*n* = 8). Two-way ANOVA followed by Bonferroni post hoc test was performed for (**a**) and (**c**) and Mann Whitney test was performed for (**b**). *p < 0.05; **p < 0.01; ***p < 0.001

CCI-injury results in severe tissue loss in the ipsilateral motor cortex and substantial damage to the underlying hippocampus. To access the motor and cognition deficit, we performed beam balance test and NOR test, respectively. In the beam balance test, motor function was scored on the basis of the falling latency and the turning and moving pattern. Before CCI injury, motor function was indistinguishable in all groups, but when assessed at 9 days after CCI injury, the CCI group showed reduced performance (Fig. 8a). Notably, systemic administration of G1023 to CCI group significantly improved performance on the beam as compared to vehicle-treated CCI group (Fig. 8a).

The possible effect of G1023 on cognition or memory was examined in the NOR test (Fig. 8b–d), which exploits the natural preference for novel objects displayed by mice. During habituation, no significant differences were observed in the total exploration time among groups, when mice were exposed to two familiar objects. When the mice were exposed to one familiar and one novel object, vehicle-sham and G1023-sham groups approached frequently and spent more time exploring the novel than the familiar one (Fig. 8c). However, CCI group spent much less time in exploring the novel object than the two control groups, as indicated in the lower DI. G1023 treatment markedly improved the DI value of CCI group (Fig. 8d). The results from beam balance test and NOR test suggest that G1023 improves neurobehavioral outcomes following CCI.

## Discussion

*LRRK*2 mRNA is widely detected throughout the brain and other organs^43,44^ and its expression seem to change during development, as the animal ages, and in specific pathological conditions^17,43,45,46^. However, surprisingly little is known about the mechanism by which endogenous, wild-type LRRK2 is regulated. Understanding the cues and the regulatory mechanism of LRRK2 expression are likely to provide insights into the role of LRRK2 in physiological and pathological conditions. Here we show that various types of injuries induce LRRK2 expression in neurons, and we provide evidence for HIF-1α-dependent transcriptional regulation of LRRK2 expression.

In immune cells, various pathological stimuli increase LRRK2 level, but the molecular mechanism of LRRK2 induction remains largely unexplored. A recent study showed that LRRK2 level increased in neurons and microglia after weight drop brain injury in rats^47^, but the precise mechanism and the physiological significance of the increase have remained unclear. Here, we show that neurons robustly increase LRRK2 expression in response to injury and such increase was also detected in postmortem brains of CTE patients. Brain injury is often accompanied by glutamate excitotoxicity, oxidative stress, hypoxia, and neuroinflammation, and thus diverse molecular pathways might contribute to the induction of LRRK2. Given that diverse signals potently induced LRRK2 expression and neurotoxicity in primary neuronal cultures, LRRK2 induction might not necessarily require other cell types in the brain. Secreted factors from damaged neurons might play a part because conditioned media from scratch-injured neurons was sufficient to induce LRRK2 expression and neurotoxicity in intact neurons.

*In silico* analysis of the human *LRRK2* promoter revealed multiple putative binding sites for several transcription factors, such as Sp-1, c-Jun, HNF-3α, GATA-1/2, NFAT, EGF1/2/3, NRSE, MEF-1, and IRF^17,48,49^. However, with the exception of Sp-1, most sites await further biochemical and functional validations^48^. Here we show that injury increases both LRRK2 and HIF-1α levels and that HIF-1α regulates LRRK2 expression. In the promoter region of *LRRK2*, we found two sense and two anti-sense HRE consensus sequences that can recruit the HIF-1α/β heterodimer^50,51^. Although HRE consensus sequences are frequently found across the genome, less than 1% of the potential sites are estimated to actually bind HIFs in response to hypoxia^50,52^. Through luciferase reporter assay, mutagenesis, and ChIP assay, we confirmed direct binding of HIF-1α to HRE3 in mouse *LRRK2* promoter and validated that HRE3 is functional. Although sequence homology between mouse and human *LRRK2* promoters was low, multiple HRE sequences were also identified in the human *LRRK2* promoter and we confirmed that human *LRRK2* expression was also regulated by HIF-1α.

The identification of HIF-1α-LRRK2 axis implies that LRRK2 may be involved in a number of physiological and pathological conditions regulated by HIF-1α. In the brain, HIF-1 has been implicated in a wide range of pathological conditions, such as cerebral ischemia^53^, TBI^54^, and neurodegenerative diseases^55–57^, presumably through transcriptional regulation of downstream target genes. Gene products of HIF-1 induction following brain injury can lead to neuroprotective or detrimental effects depending on the type of cellular stress and severity of insults, which would affect the duration and extent of HIF-1 activation as well as the activity of its up- and downstream regulators^58,59^. Given the prominent neuronal cell death induced by TBI, it is plausible that TBI-induced activation of the HIF-1α-LRRK2 axis triggers key molecules in pro-apoptotic pathways^38^. LRRK2 kinase domain contains sequence homology to mixed-lineage kinase (MLK) subfamily of MAPKKK. MLKs can be simulated by various stress stimuli and activate cell death pathways by phosphorylating downstream MKKs^60^. LRRK2 has been shown to phosphorylate MKK3, 4, 6, and 7^61^, and the loss of dopaminergic neurons in LRRK2-G2019S transgenic mice has been attributed to the activation of MKK4-JNK-c-Jun pathway^41^. A recent study showed that activation of ASK1 by LRRK2 triggered MKK3/6-p38 MAPK signaling^62^. Consistent with previous studies, here we suggest that LRRK2 activation induced by injury causes cell death via triggering the ASK1-MKK-JNK/c-Jun pathway.

Over the last several decades, phase III clinical trials in TBI have met with limited success^25^. Since TBI is a highly complex and heterogeneous disorder, TBI should be treated by combining multiple agents designed to have complementary effects or using a multipotent agent that can simultaneously control multiple injury mechanisms. In this regard, LRRK2 represents an attractive target because LRRK2 inhibition can prevent the activation of neuronal cell death pathways and suppress inflammation. Here we show that brain injury increased LRRK2 expression in microglia and that inflammatory responses, such as recruitment of activated microglia and the expression of pro-inflammatory cytokines (*IL-1β*, *IL-6*, and *TNF-α*), induced by CCI were all reduced after systemic administration of LRRK2 inhibitor. It is likely that LRRK2 inhibitors suppressed neuroinflammation by directly acting on innate immunity or through an indirect mechanism via attenuating neuronal cell death or a combination of both.

In conclusion, the present study identifies a previously undefined HIF-1α-dependent transcriptional regulatory mechanism of LRRK2 expression and a neuropathological consequence of LRRK2 induction after TBI. Our findings suggest a possibility of a significant extension of LRRK2 function in a wide range of pathologies and human diseases in which HIF-1 is involved. Future studies are needed to investigate if regulation of the HIF1-α-LRRK2 axis has a therapeutic potential in such pathologies.

## Electronic supplementary material

Supplemental informatin

## References

[1] Biskup S (2006). Localization of LRRK2 to membranous and vesicular structures in mammalian brain. Ann. Neurol..

[2] Han BS (2008). Expression of the LRRK2 gene in the midbrain dopaminergic neurons of the substantia nigra. Neurosci. Lett..

[3] Zimprich A (2004). Mutations in LRRK2 cause autosomal-dominant parkinsonism with pleomorphic pathology. Neuron.

[4] Di Fonzo A (2005). A frequent LRRK2 gene mutation associated with autosomal dominant Parkinson’s disease. Lancet.

[5] Gilks WP (2005). A common LRRK2 mutation in idiopathic Parkinson’s disease. Lancet.

[6] Lee BD (2010). Inhibitors of leucine-rich repeat kinase-2 protect against models of Parkinson’s disease. Nat. Med..

[7] Greggio E (2006). Kinase activity is required for the toxic effects of mutant LRRK2/dardarin. Neurobiol. Dis..

[8] Smith WW (2006). Kinase activity of mutant LRRK2 mediates neuronal toxicity. Nat. Neurosci..

[9] Jeong GR (2018). Dysregulated phosphorylation of Rab GTPases by LRRK2 induces neurodegeneration. Mol. Neurodegener..

[10] Migheli R (2013). LRRK2 affects vesicle trafficking, neurotransmitter extracellular level and membrane receptor localization. PLoS. One..

[11] Matta S (2012). LRRK2 controls an EndoA phosphorylation cycle in synaptic endocytosis. Neuron.

[12] Habig K (2013). LRRK2 guides the actin cytoskeleton at growth cones together with ARHGEF7 and Tropomyosin 4. Biochim. Biophys. Acta.

[13] Martin I (2014). Ribosomal protein s15 phosphorylation mediates LRRK2 neurodegeneration in Parkinson’s disease. Cell.

[14] Orenstein SJ (2013). Interplay of LRRK2 with chaperone-mediated autophagy. Nat. Neurosci..

[15] Alegre-Abarrategui J (2009). LRRK2 regulates autophagic activity and localizes to specific membrane microdomains in a novel human genomic reporter cellular model. Hum. Mol. Genet..

[16] Niu J, Yu M, Wang C, Xu Z (2012). Leucine-rich repeat kinase 2 disturbs mitochondrial dynamics via Dynamin-like protein. J. Neurochem..

[17] Gardet A (2010). LRRK2 is involved in the IFN-gamma response and host response to pathogens. J. Immunol..

[18] Hakimi M (2011). Parkinson’s disease-linked LRRK2 is expressed in circulating and tissue immune cells and upregulated following recognition of microbial structures. J. Neural Transm..

[19] Zhang FR (2009). Genomewide association study of leprosy. N. Engl. J. Med..

[20] Barrett JC (2008). Genome-wide association defines more than 30 distinct susceptibility loci for Crohn’s disease. Nat. Genet..

[21] Thevenet J, Pescini Gobert R, Hooft van Huijsduijnen R, Wiessner C, Sagot YJ (2011). Regulation of LRRK2 expression points to a functional role in human monocyte maturation. PLoS. One..

[22] Kuss M, Adamopoulou E, Kahle PJ (2014). Interferon-gamma induces leucine-rich repeat kinase LRRK2 via extracellular signal-regulated kinase ERK5 in macrophages. J. Neurochem..

[23] Moehle MS (2012). LRRK2 inhibition attenuates microglial inflammatory responses. J. Neurosci..

[24] Gillardon F, Schmid R, Draheim H (2012). Parkinson’s disease-linked leucine-rich repeat kinase 2(R1441G) mutation increases proinflammatory cytokine release from activated primary microglial cells and resultant neurotoxicity. Neuroscience.

[25] Loane DJ, Faden AI (2010). Neuroprotection for traumatic brain injury: translational challenges and emerging therapeutic strategies. Trends Pharmacol. Sci..

[26] McAllister TW (2011). Neurobiological consequences of traumatic brain injury. Dialog-. Clin. Neurosci..

[27] Maas AI (2010). Common data elements for traumatic brain injury: recommendations from the interagency working group on demographics and clinical assessment. Arch. Phys. Med. Rehabil..

[28] Werner C, Engelhard K (2007). Pathophysiology of traumatic brain injury. Br. J. Anaesth..

[29] Cruz-Haces M, Tang J, Acosta G, Fernandez J, Shi R (2017). Pathological correlations between traumatic brain injury and chronic neurodegenerative diseases. Transl. Neurodegener..

[30] Kumar A, Loane DJ (2012). Neuroinflammation after traumatic brain injury: opportunities for therapeutic intervention. Brain Behav. Immun..

[31] Palmer AM (1993). Traumatic brain injury-induced excitotoxicity assessed in a controlled cortical impact model. J. Neurochem..

[32] Choi WW (2017). The effects of Chunghyul-Dan, an agent of Korean medicine, on a mouse model of traumatic brain injury. Evid. Based Complement. Altern. Med..

[33] Villapol S, Balarezo MG, Affram K, Saavedra JM, Symes AJ (2015). Neurorestoration after traumatic brain injury through angiotensin II receptor blockage. Brain.

[34] Luo P (2014). Postsynaptic scaffold protein Homer 1a protects against traumatic brain injury via regulating group I metabotropic glutamate receptors. Cell Death Dis..

[35] Han Z (2014). miR-21 alleviated apoptosis of cortical neurons through promoting PTEN-Akt signaling pathway in vitro after experimental traumatic brain injury. Brain Res..

[36] Yin J (2017). Transglutaminase 2 inhibition reverses mesenchymal transdifferentiation of glioma stem cells by regulating C/EBPbeta signaling. Cancer Res..

[37] Osier ND, Dixon CE (2016). The controlled cortical impact model: applications, considerations for researchers, and future directions. Front. Neurol..

[38] Li A, Sun X, Ni Y, Chen X, Guo A (2013). HIF-1alpha involves in neuronal apoptosis after traumatic brain injury in adult rats. J. Mol. Neurosci..

[39] Guzy RD (2005). Mitochondrial complex III is required for hypoxia-induced ROS production and cellular oxygen sensing. Cell. Metab..

[40] Mabjeesh NJ (2003). 2ME2 inhibits tumor growth and angiogenesis by disrupting microtubules and dysregulating HIF. Cancer Cell..

[41] Chen CY (2012). (G2019S) LRRK2 activates MKK4-JNK pathway and causes degeneration of SN dopaminergic neurons in a transgenic mouse model of PD. Cell Death Differ..

[42] Sheng Z (2012). Ser1292 autophosphorylation is an indicator of LRRK2 kinase activity and contributes to the cellular effects of PD mutations. Sci. Transl. Med.

[43] Biskup S (2007). Dynamic and redundant regulation of LRRK2 and LRRK1 expression. Bmc. Neurosci..

[44] Melrose HL (2007). A comparative analysis of leucine-rich repeat kinase 2 (Lrrk2) expression in mouse brain and Lewy body disease. Neuroscience.

[45] Maekawa T, Kubo M, Yokoyama I, Ohta E, Obata F (2010). Age-dependent and cell-population-restricted LRRK2 expression in normal mouse spleen. Biochem. Biophys. Res. Commun..

[46] Cook DA (2017). LRRK2 levels in immune cells are increased in Parkinson’s disease. NPJ Park. Dis..

[47] Rui Q (2018). LRRK2 contributes to secondary brain injury through a p38/Drosha signaling pathway after traumatic brain injury in rats. Front. Cell. Neurosci..

[48] Wang J, Song W (2016). Regulation of LRRK2 promoter activity and gene expression by Sp1. Mol. Brain.

[49] West AB (2005). Parkinson’s disease-associated mutations in leucine-rich repeat kinase 2 augment kinase activity. Proc. Natl. Acad. Sci. USA.

[50] Schodel J (2011). High-resolution genome-wide mapping of HIF-binding sites by ChIP-seq. Blood.

[51] Dengler VL, Galbraith M, Espinosa JM (2014). Transcriptional regulation by hypoxia inducible factors. Crit. Rev. Biochem. Mol. Biol..

[52] Mole DR (2009). Genome-wide association of hypoxia-inducible factor (HIF)-1alpha and HIF-2alpha DNA binding with expression profiling of hypoxia-inducible transcripts. J. Biol. Chem..

[53] Wiener CM, Booth G, Semenza GL (1996). In vivo expression of mRNAs encoding hypoxia-inducible factor 1. Biochem. Biophys. Res. Commun..

[54] Yu R, Gao L, Jiang S, Guan P, Mao B (2001). Association of HIF-1alpha expression and cell apoptosis after traumatic brain injury in the rat. Chin. J. Traumatol..

[55] Avramovich-Tirosh Y, Bar-Am O, Amit T, Youdim MB, Weinreb O (2010). Up-regulation of hypoxia-inducible factor (HIF)-1alpha and HIF-target genes in cortical neurons by the novel multifunctional iron chelator anti-Alzheimer drug, M30. Curr. Alzheimer Res..

[56] Lee DW (2009). Inhibition of prolyl hydroxylase protects against 1-methyl-4-phenyl-1,2,3,6-tetrahydropyridine-induced neurotoxicity: model for the potential involvement of the hypoxia-inducible factor pathway in Parkinson disease. J. Biol. Chem..

[57] Zhang Z, Yan J, Chang Y, ShiDu Yan S, Shi H (2011). Hypoxia inducible factor-1 as a target for neurodegenerative diseases. Curr. Med. Chem..

[58] Yeh SH, Ou LC, Gean PW, Hung JJ, Chang WC (2011). Selective inhibition of early—but not late—expressed HIF-1alpha is neuroprotective in rats after focal ischemic brain damage. Brain. Pathol..

[59] Shi H (2009). Hypoxia inducible factor 1 as a therapeutic target in ischemic stroke. Curr. Med. Chem..

[60] Gallo KA, Johnson GL (2002). Mixed-lineage kinase control of JNK and p38 MAPK pathways. Nat. Rev. Mol. Cell Biol..

[61] Gloeckner CJ, Schumacher A, Boldt K, Ueffing M (2009). The Parkinson disease-associated protein kinase LRRK2 exhibits MAPKKK activity and phosphorylates MKK3/6 and MKK4/7, in vitro. J. Neurochem..

[62] Yoon JH (2017). LRRK2 functions as a scaffolding kinase of ASK1-mediated neuronal cell death. Biochim. Biophys. Acta.

