# Supplementary material for: Brain injury induces HIF-1α-dependent transcriptional activation of LRRK2 that exacerbates brain damage
Source: Cell Death Dis. 2018 Nov 12;9(11):1125. doi: 10.1038/s41419-018-1180-y (PMC6232134; doi:10.1038/s41419-018-1180-y)
Supplement: Supplementary file 1 — Supplemental informatin [file 41419_2018_1180_MOESM1_ESM.pdf]

Title: **Brain injury induces HIF-1α-dependent transcriptional activation of LRRK2 that exacerbates brain damage**

Bae et. al.

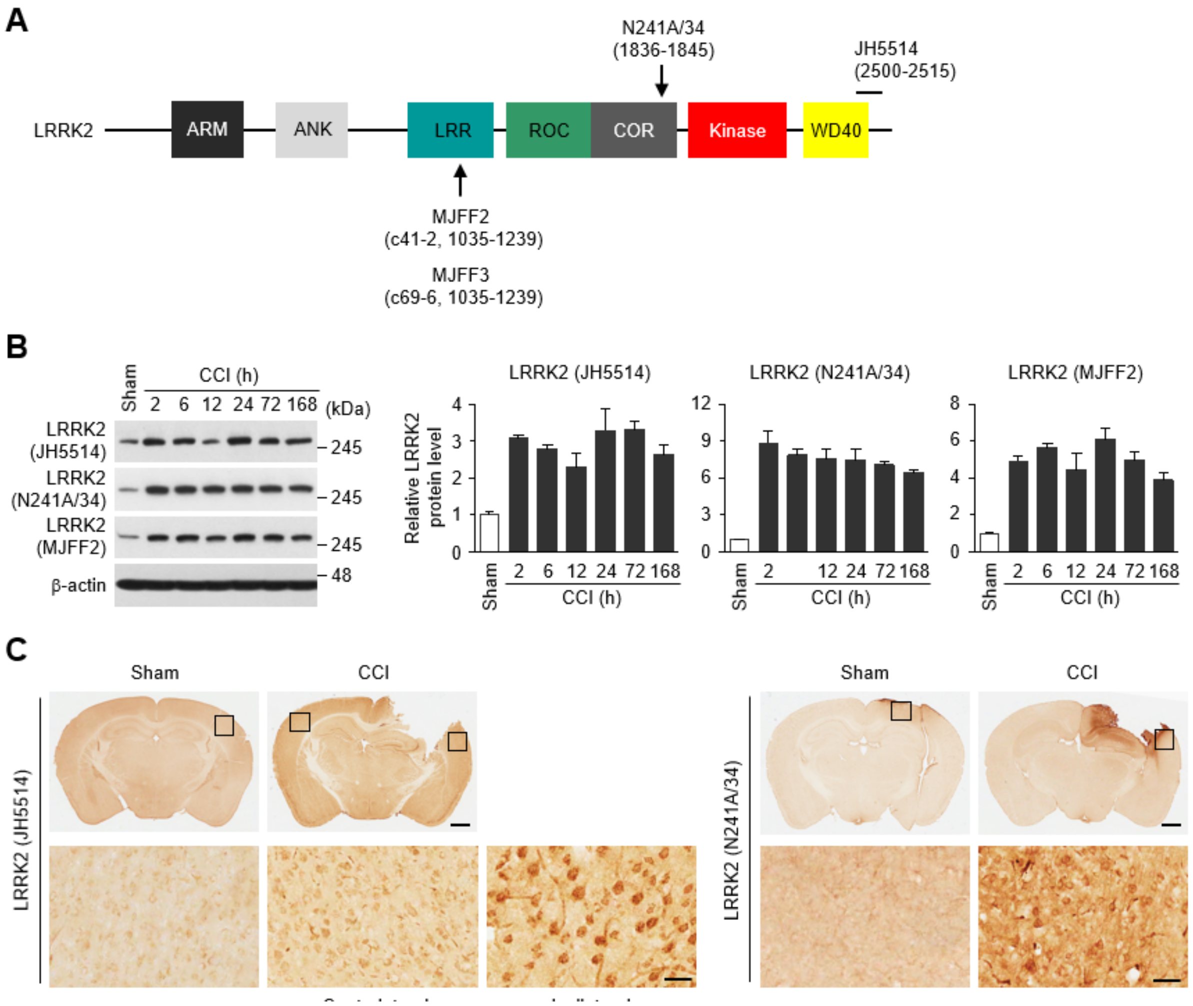

Supplementary figure 1. LRRK2 induction in *in vivo* TBI model. (A) Schematic diagram indicating antigen information of LRRK2 antibodies used in this paper. (B) Level of LRRK2 protein in brain lysates from the ipsilateral side of sham and CCI group. Shown are representative blots (B, *left*) and quantification of LRRK2 protein levels (B, *right*) relative to sham. Bar graph shows means ± s.d. (n=3). (C) LRRK2 immunostaining from coronal brain sections of sham and CCI group at 24 hr post-injury. Magnified images of indicated area in *upper rows* are presented in *lower row*. Scale bar = 1 mm (*upper row*) and 50 µm (*lower row*).

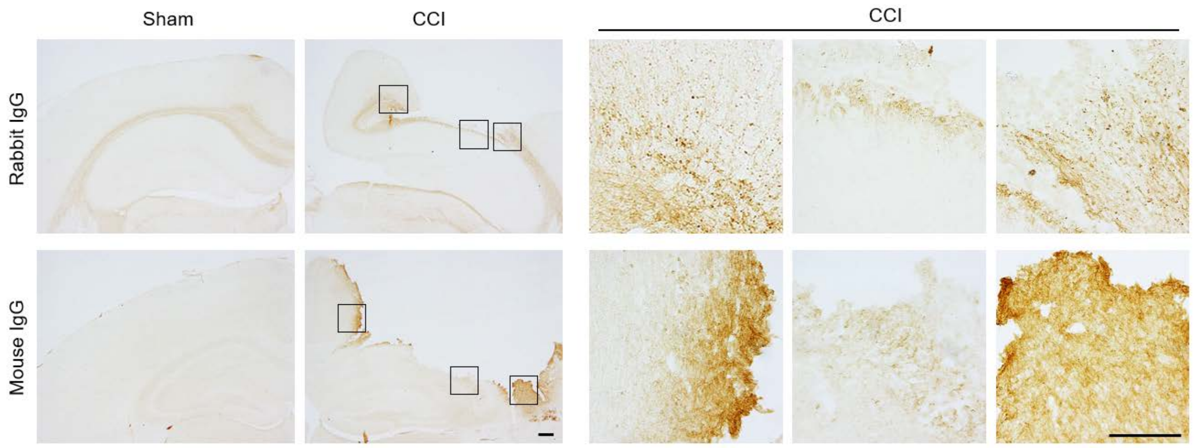

Supplementary figure 2. Immunostaining with control IgG antibodies. Immunostaining of sham and CCI brain sections collected at 24 hr post-injury with rabbit IgG or mouse IgG control antibodies. Magnified images of indicated area in *left* are presented in *right*. Scale bar = 200 μm.

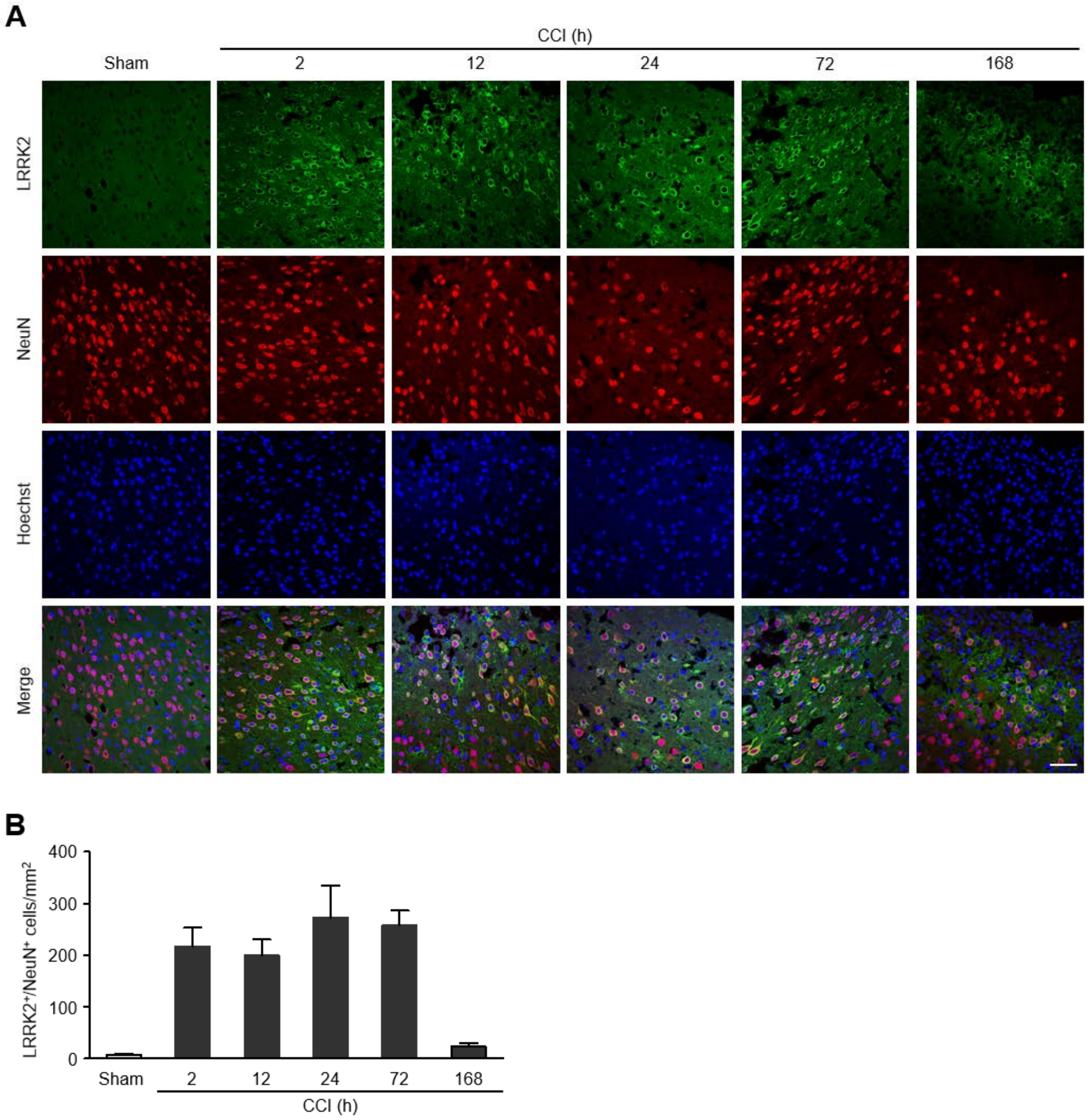

Supplementary figure 3. LRRK2 induction in neuron after CCI injury. Co-immunostaining with LRRK2 and NeuN antibodies from coronal brain sections of sham and CCI group. (A) Representative image in pericontusion region of CCI group and corresponding region of sham group immunostained with LRRK2 and NeuN antibodies. Scale bar = 50 μm. (B) Numbers of LRRK2 and NeuN double immuno-positive cells (per mm^2). Bar graph shows means ± s.d. (n=3).

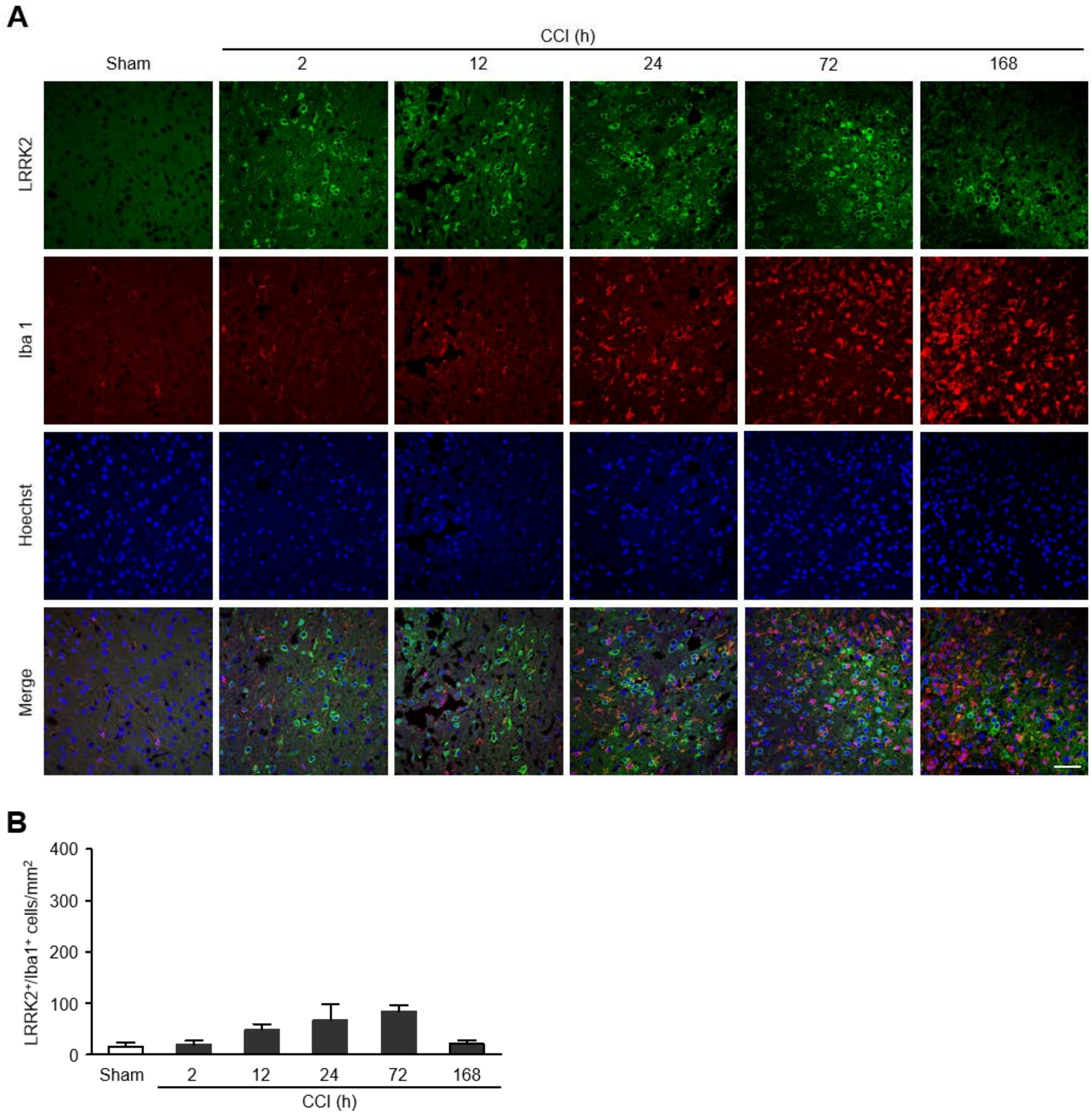

Supplementary figure 4. LRRK2 induction in microglia after CCI injury. Co-immunostaining with LRRK2 and Iba1 antibodies from coronal brain sections of sham and CCI group. (A) Representative image in pericontusion region of CCI group and corresponding region of sham group immunostained with LRRK2 and Iba1 antibodies. Scale bar = 50 μm. (B) Numbers of LRRK2 and Iba1 double immuno-positive cells (per mm^2). Bar graph shows means ± s.d. (n=3).

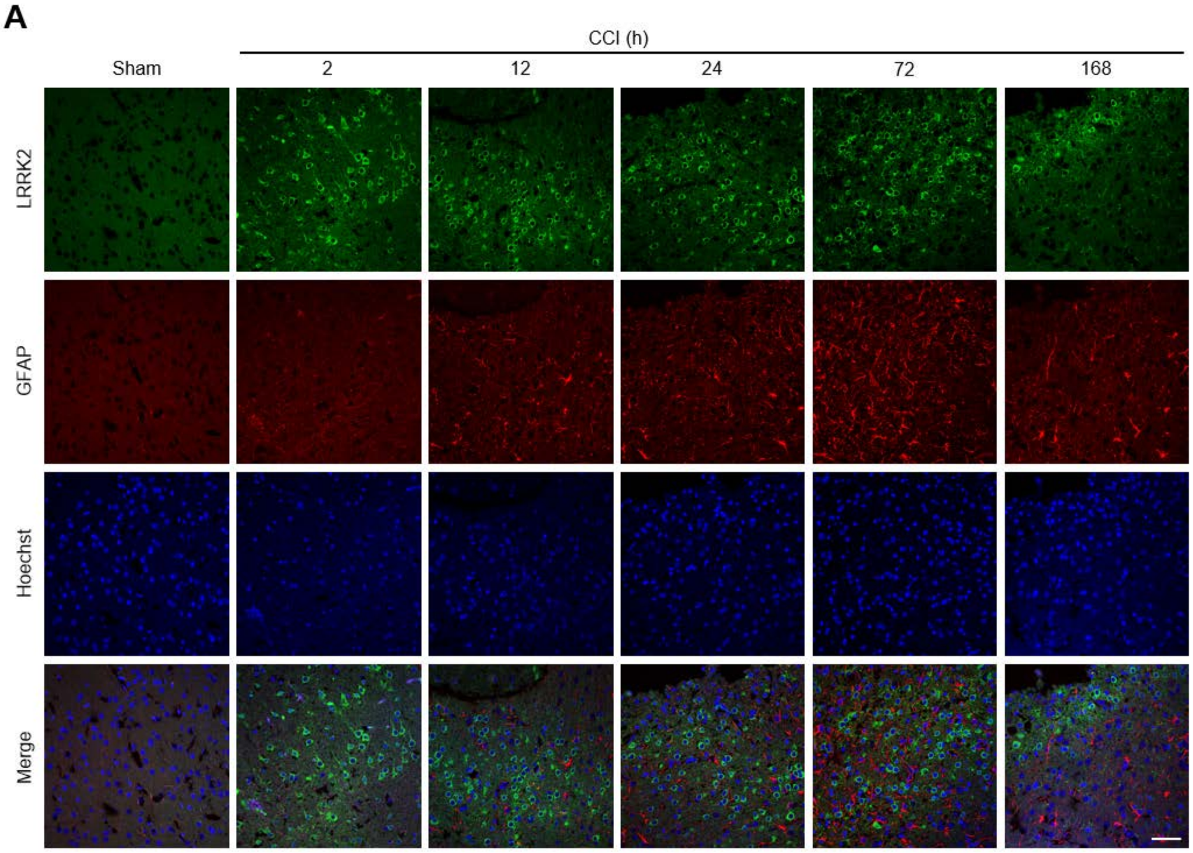

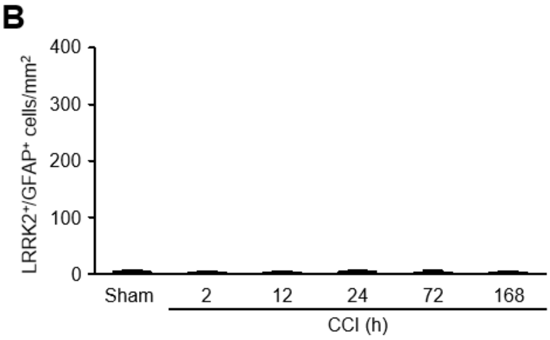

Supplementary figure 5. LRRK2 induction in astrocyte after CCI injury. Co-immunostaining with LRRK2 and GFAP antibodies from coronal brain sections of sham and CCI group. (a) Representative image in pericontusion region of CCI group and corresponding region of sham group immunostained with LRRK2 and GFAP antibodies. Scale bar = 50 μm. (b) Numbers of LRRK2 and GFAP double immuno-positive cells (per mm^2). Bar graph shows means ± s.d. (n=3).

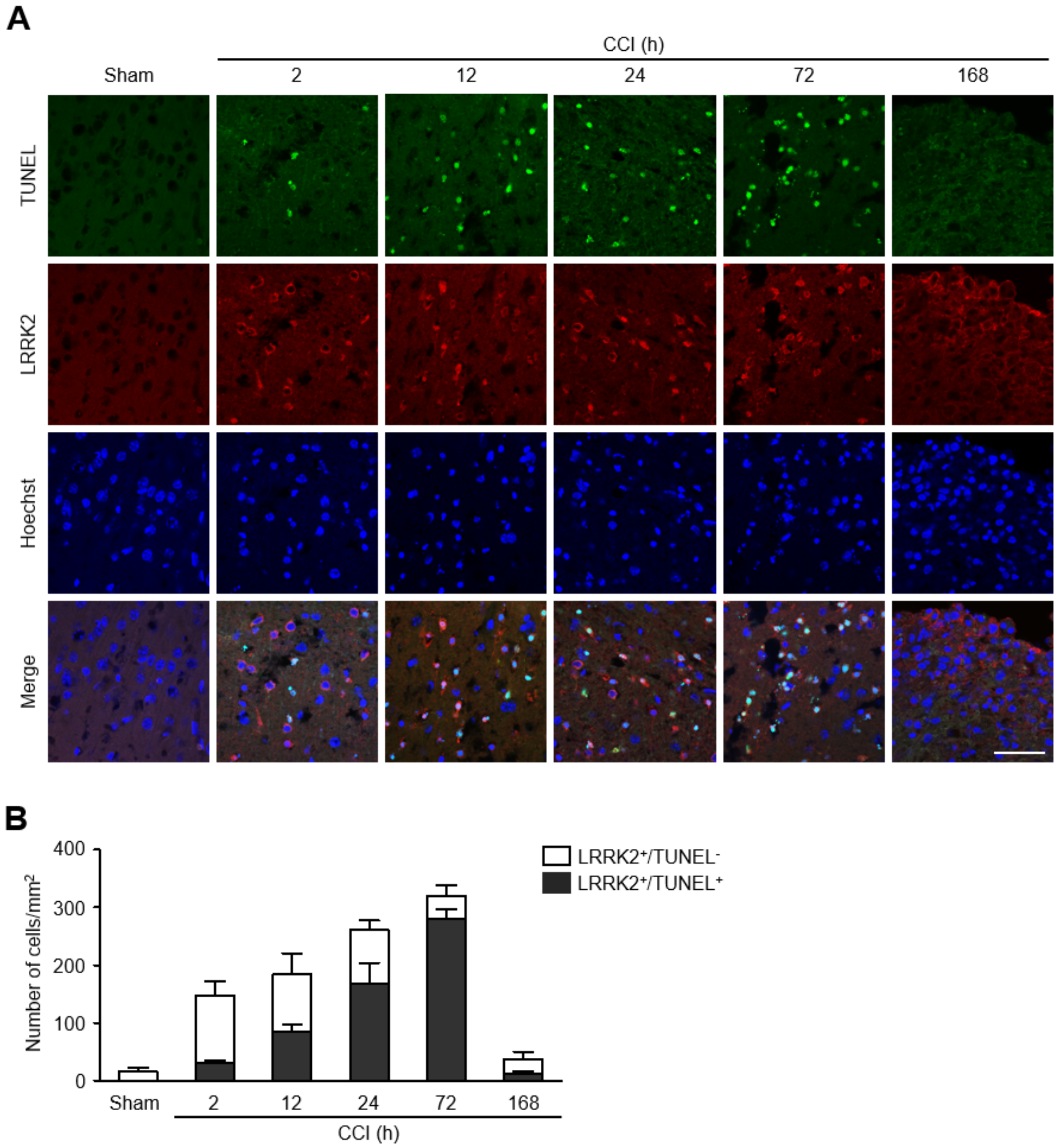

Supplementary figure 6. Apoptosis in LRRK2 induced cells after CCI injury. TUNEL staining and LRRK2 immunostaining from coronal brain sections of sham and CCI group. (A) Representative TUENL image in pericontusion region of CCI group and corresponding region of sham group. Scale bar = 50 μm. (B) Numbers of $LRRK2^+/TUNEL^+$ and $LRRK2^+/TUNEL^-$ cells (per mm^2). Bar graph shows means ± s.d. (n=3).

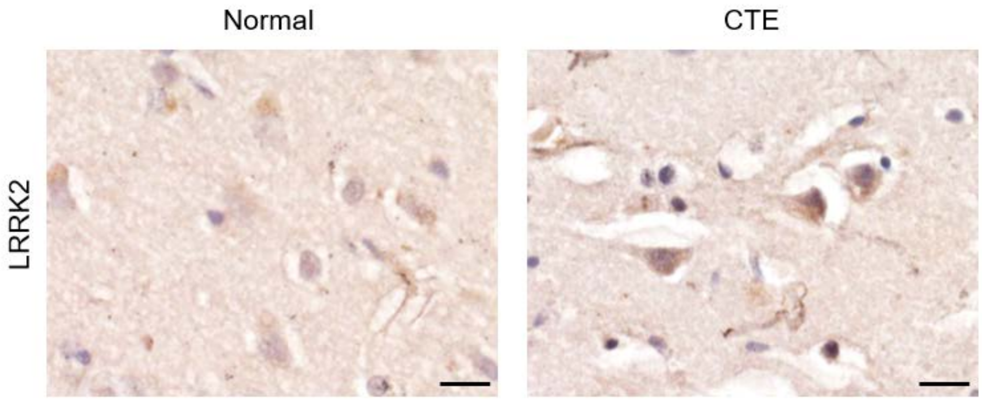

Supplementary figure 7. LRRK2 induction in the frontal cortex of CTE patients. Representative images of LRRK2 immunostaining in postmortem brain section from normal subject and CTE patient. Scale bar = 20 μm.

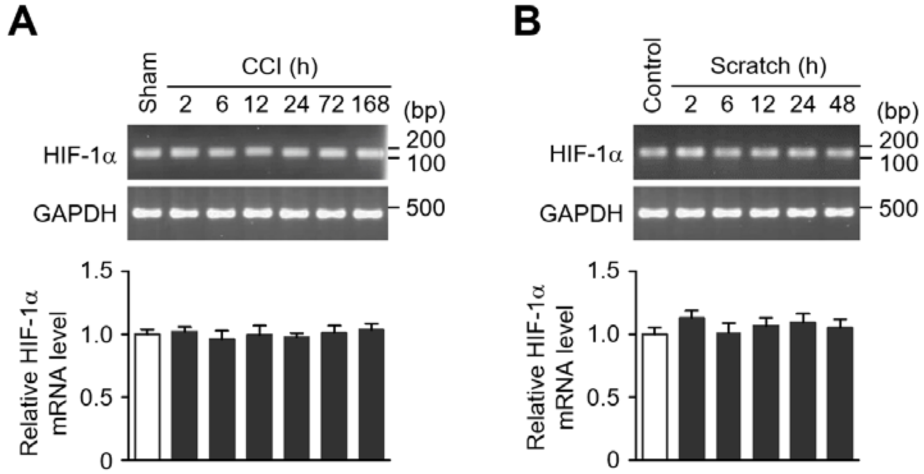

Supplementary figure 8. The level of *HIF-1α* mRNA in *in vivo* and *in vitro* TBI models. (A) The level of *HIF-1α* mRNA in Sham and CCI brains. Shown are representative gel images (A, *top*) and quantification of *HIF-1α* mRNA (A, *bottom*) relative to sham. Bar graph shows means ± s.d. (n=3). (B) The level of *HIF-1α* mRNA in control and scratch injured cortical neurons. Shown are representative gel images (B, *top*) and quantification of *HIF-1α* mRNA (B, *bottom*) relative to control. Bar graph shows means ± s.d. (n=3).

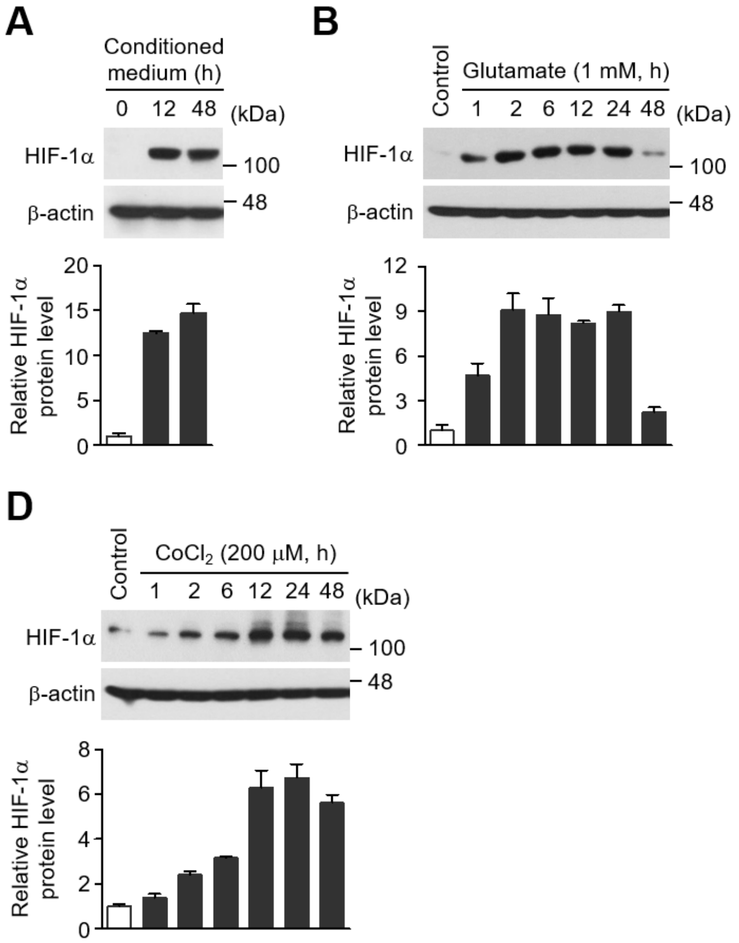

Supplementary figure 9. HIF-1α induction after treatment of neuronal toxic assaults. Levels of HIF-1α in cortical neurons treated with conditioned medium from scratch-injured cortical neurons (A), 1 mM glutamate (B), 100 μM H_2O_2 (C), and 200 μM $CoCl_2$ (D). Shown are representative immunoblots (*top*) and quantification of LRRK2 protein level (*bottom*). Bar graph shows mean ± s.d. (n=3).

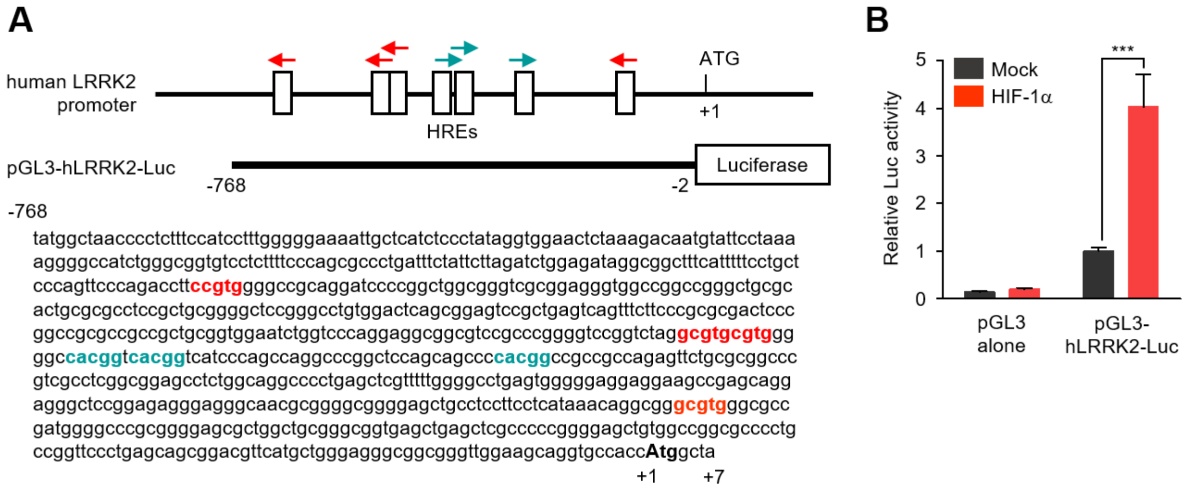

Supplementary figure 10. Transcriptional regulation of human *LRRK2* by HIF-1α. (A) Putative HRE sites in human *LRRK2* promoter and promoter sequence. (B) pGL3-hLRRK2-Luc and pcDNA3-HA-HIF-1α-WT were co-transfected into human neuroblastoma SH-SY5Y cells and luciferase activity was measured after 48 hr. Bar graph shows means ± s.d. (n=3). One-way ANOVA followed by Newman-Keuls post hoc test was performed. ***p < 0.001.

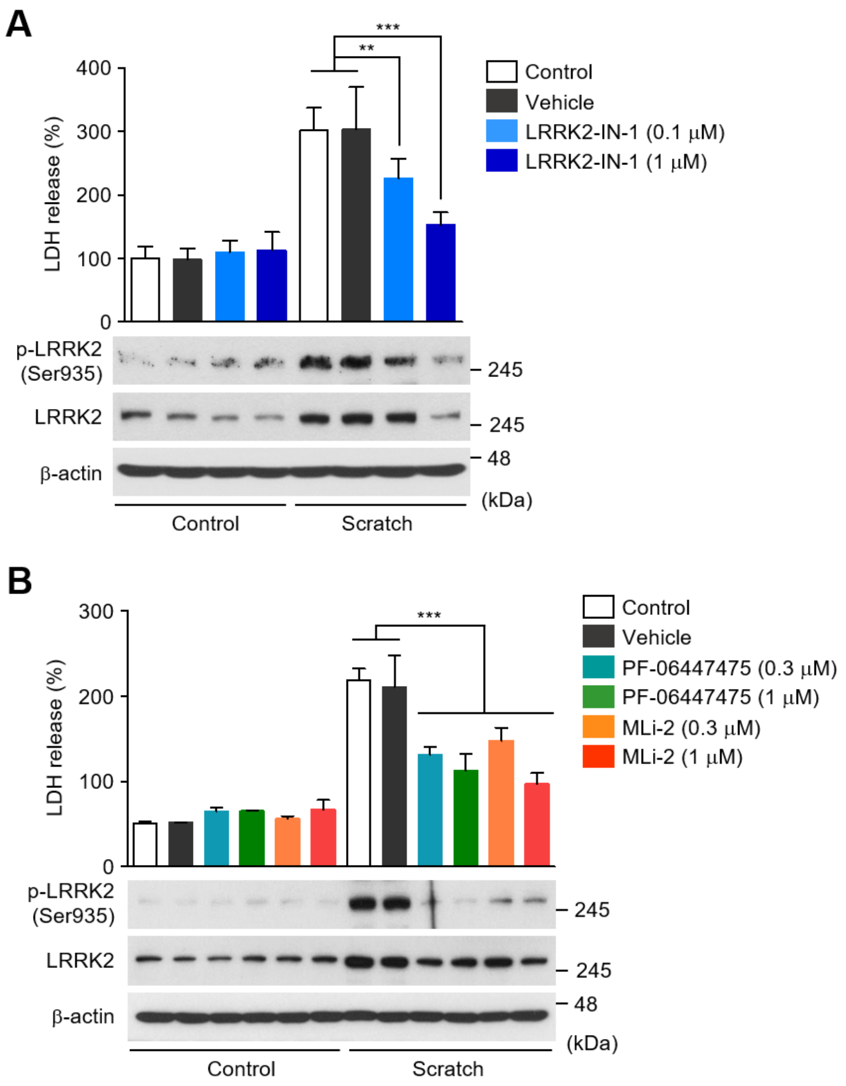

Supplementary figure 11. The effect of LRRK2 kinase inhibitors on scratch induced neuronal toxicity. Cortical neurons were pretreated with vehicle (0.05% DMSO) or LRRK2 kinase inhibitors, (A) LRRK2-IN-1 (0.1 and 1 μM), (B) PF-06447475 (0.3 and 1 μM), or (B) MLi-2 (0.3 and 1 μM)) for 1 hr prior to scratch injury at DIV 10. After 48 hrs, the analyses described below were performed. LDH release assay (*top*). LDH release level relative to control neurons treated with vehicle control. Bar graph shows means ± s.d. (n=3). Levels of total and phospho-S935 LRRK2 protein in control and scratch-injured cortical neurons treated with LRRK2-IN-1, PF-06447475, MLi-2, or vehicle control were measured. Representative immunoblots (bottom) are shown. One-way ANOVA followed by Newman-Keuls post hoc test was performed for all experiments. $^{**}p < 0.005$, $^{***}p < 0.001$.

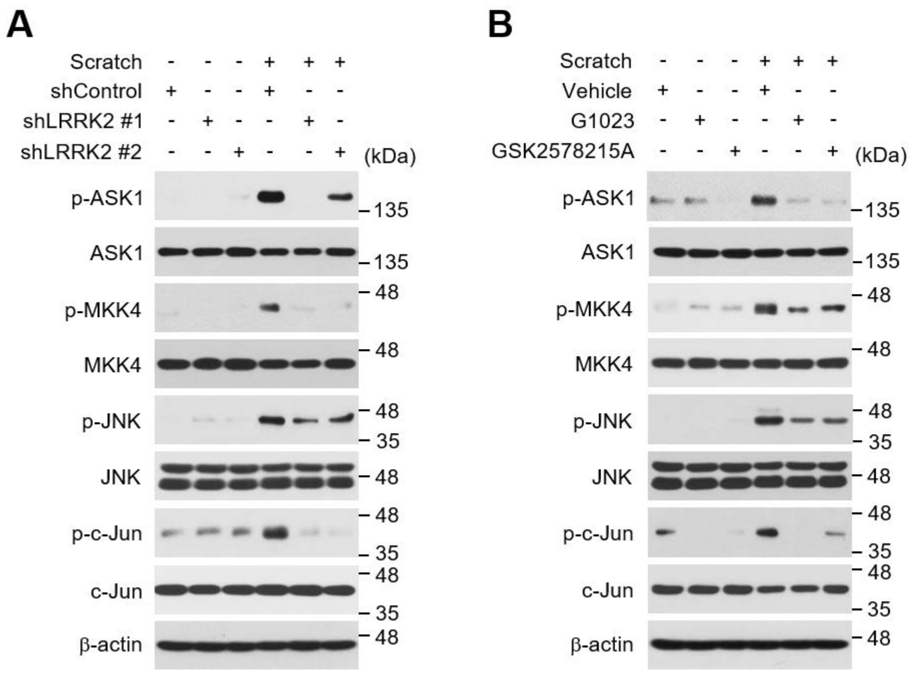

Supplementary figure 12. LRRK2 dependent regulation of AKS-1-MKK-JNK/c-Jun pathways after scratch injury. Cortical neurons were (A) infected with lentivirus (pLL3.7-shControl, shLRRK2 #1, #2) at DIV8 and (B) treated with LRRK2 kinase inhibitors, 1 μM G1023 and 1 μM GSK2578215A at DIV10. Scratch injury was applied at DIV10 and cells were harvested at 48 hr post-injury. Immunoblots were performed with indicated antibodies.

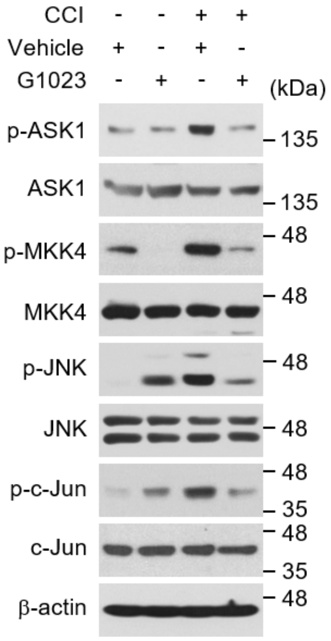

Supplementary figure 13. LRRK2 dependent regulation of AKS-1-MKK-JNK/c-Jun pathways after CCI injury. LRRK2 kinase inhibitor, G1023 was daily applied of 50 mg/kg through peritoneal injection for 3 days post-injury. Peri-contusional area of CCI group and corresponding area of sham group treated with vehicle or G1023 were collected, and subjected into immunoblots with indicated antibodies.

Supplementary Table

Supplementary Table 1. Human tissue information for normal subjects and subjects with CTE.

Case	Age	Sex	CTE stage
Normal 1	87	F	0
Normal 2	88	M	0
Normal 3	86	M	0
Normal 4	87	F	0
Normal 5	67	M	0
Normal 6	82	M	0
Normal 7	70	M	0
CTE 1	84	M	4
CTE 2	73	M	4
CTE 3	80	M	4
CTE 4	73	M	3
CTE 5	76	M	4
CTE 6	93	M	4
CTE 7	69	M	4

Supplementary Table 2. List of antibodies.

Primary antibodies				
**Antigen**	**Supplier**	**Cat. No.**	**WB**	**IHC/IF**
LRRK2	Abcam	ab133475	1:2000	
LRRK2	Abcam	ab133474	1:2000	1:300
LRRK2	Millipore	ABN187	1:2000	1:500
LRRK2	Neuromab	73-253	1:200	1:100
p-LRRK2 (Ser935)	Abcam	ab133450	1:1000	
β-actin	Abcam	ab6276	1:4000	
HIF-1α	Novus Biologicals	NB100-105	1:2000	1:250
HIF-1α	Novus Biologicals	NB100-479	1:2000	1:250
MAP2	Thermo Fisher Scientific	MA512826		1:500
MAP2	Abcam	ab32454		1:1000
Iba1	Abcam	ab5076		1:1000
GFAP	Abcam	ab7260		1:1000
GFAP	Sigma-Aldrich	G3893		1:2000
NeuN	Cell Signaling Technology	12943		1:500
NeuN	Millipore	MAB377		1:500
Cleaved Caspase-3	Cell Signaling Technology	9661	1:1000	
PARP	Cell Signaling Technology	9532	1:2000	
Bcl-2	Cell Signaling Technology	2870	1:2000	
p53	Cell Signaling Technology	2524	1:2000	
HA.11 Epitope Tag	Covance	MMS-101p-200	1:2000	
Aquaporin 4	Millipore	AB3594	1:2000	
MMP2	Santa Cruz Biotechnology	sc-80201	1:4000	
MMP9	Abcam	ab38898	1:2000	
p-SAPK/JNK (Thr183/Tyr185)	Cell Signaling Technology	9251	1:2000	
SAPK/JNK	Cell Signaling Technology	9252	1:4000	
p-c-Jun (Ser63)	Cell Signaling Technology	2361	1:2000	
c-Jun	Cell Signaling Technology	9165	1:2000	
p-SEK1/MKK4 (Ser257)	Cell Signaling Technology	4514	1:2000	
MKK4	Santa Cruz Biotechnology	sc-376838	1:2000	
p-ASK1 (Thr845)	Santa Cruz Biotechnology	sc-109911	1:2000	
ASK1	Santa Cruz Biotechnology	sc-390275	1:2000	
Mouse IgG (70 mg/ml)	Sigma-Aldrich	I5381		1:100
Rabbit IgG (100 mg/ml)	Sigma-Aldrich	I5006		1:300

Supplementary Table 2. List of antibodies (*continued*).

Secondary antibodies				
**Name**	**Supplier**	**Cat. No.**	**WB**	**IHC/IF**
anti-Mouse IgG (H+L)-HRP	Cell Signaling Technology	7076	1:5000	
anti-Rabbit IgG (H+L)-HRP	Cell Signaling Technology	7074	1:5000	
anti-Goat IgG (whole molecule)-HRP	Sigma-Aldrich	A5420	1:5000	
anti-Mouse IgG (H+L) -Biotin	KPL	16-18-15		1:500
anti-Rabbit IgG (H+L) -Biotin	Vector Laboratories	BA-1000		1:500
anti-Goat IgG (H+L)-Biotin	Vector Laboratories	BA-5000		1:500
anti-Rabbit IgG (H+L) -Alexa Fluor 488	Thermo Fisher Scientific	A11034		1:500
anti-Rabbit IgG (H+L) -Alexa Fluor 568	Thermo Fisher Scientific	A10042		1:500
anti-Mouse IgG (H+L) -Alexa Fluor 488	Thermo Fisher Scientific	A11029		1:500
anti-Mouse IgG (H+L) -Alexa Fluor 568	Thermo Fisher Scientific	A10037		1:500
anti-Goat IgG (H+L) -Alexa Fluor 594	Vector Laboratories	A21468		1:500

Supplementary Table 3. List of oligomer sequences.

Name		Primer sequences	Product (bp)	Genbank accession number
Mouse gene-specific primers used for RT-PCR analysis (Fig. 1a, 2a, 3g, 3j, 7e, and Supplementary Fig. 8)				
GAPDH	Forward	5'-GTCATCATCTCCGCCCCTTCTGC-3'	443	AK140794
	Reverse	5'-GATGCCTGCTTCACCACCTTCTTG-3'		
LRRK2	Forward	5'-AGCTGGTTCTAGTCGCTCTG-3'	310	AY792512
	Reverse	5'-GTAGTCTGCACCTCAGCAAC-3'		
HIF-1α	Forward	5'-CTCATCAGTTGCCACTTCC-3'	126	AF003695
	Reverse	5'-TCATCTTCACTGTCTAGACCAC-3'		
IL-1β	Forward	5'-TCCAGGATGAGGACATGAGCAC-3'	105	M15131
	Reverse	5'-GAACGTCACACACCAGCAGGTTA-3'		
IL-6	Forward	5'-CCACTTCACAAGTCGGAGGCTTA-3'	169	X54542
	Reverse	5'-CCAGTTTGGTAGCATCCATCATTTC-3'		
TNF-α	Forward	5'-CTTCTGTCTACTGAACTTCGG-3'	134	X02611
	Reverse	5'-CAGGCTTGTCACTCGAATTTT-3'		
Human gene-specific primers used for qPCR analysis (Supplementary Fig. 3)				
LRRK2	Forward	5'-GAGGCGCTTCGAGCTATTT-3'		AY792511
	Reverse	5'-CTGAATCCCAGGATTTCCAA-3'		
GAPDH	Forward	5'-GAAATCCCATCACCATCTTCC-3'		M133197
	Reverse	5'-GAGGCTGTTGTCATACTTCTC-3'		
shRNA oligonucleotides for mouse LRRK2 (Fig. 5a-5f and Supplementary Fig. 12a)				
shControl	Forward	5'-T**CGCTGAGTACTTCGAAATGTC**TTCAAGAGA**GACATTTCGAAGTACTCAGCG**TTTTTTC-3'		
	Reverse	5'-TCGAGAAAAAA**CGCTGAGTACTTCGAAATGTC**TCTCTTGAA**GACATTTCGAAGTACTCAGCG**A-3'		
shLRRK2 #1	Forward	5'-T**GCTTACTACTTCACGATATTT**TTCAAGAGAAAATATCGTGAAGTAGTAAGCTTTTTTC-3'		AY792512
	Reverse	5'-TCGAGAAAAAA**GCTTACTACTTCACGATATTT**TCTCTTGAA**AAATATCGTGAAGTAGTAAGC**A-3'		
shLRRK2 #2	Forward	5'-TAAGTTGATAGTCAGGCTGAATTTCAAGAGAATTCAGCCTGACTATCAACTTTTTTTTC-3'		AY792512
	Reverse	5'-TCGAGAAAAAA**AAGTTGATAGTCAGGCTGAAT**TCTCTTGAA**ATTCAGCCTGACTATCAACTT**A-3'		
Primers used for dominant negative human HIF-1a construct (Fig. 3g-3i and 4c)[#]				
HA-DN-hHIF-1α-pcDNA3	Forward	5'-*GGGATCC* CGGCGAAGTAAAGAATCT-3'		
	Reverse	5'-*GGATATC* TCATTTGTCAAAGACGCTACT-3'		
Primers used for mouse and human LRRK2 promoter constructs (Fig. 4 and Supplementary Fig. 10)				
pGL3-mLRRK2-Luc	Forward	5'-GGGTACCGCTGTGGTTGGCCATGTCAG-3'		AC099704
	Reverse	5'-GCTCGAGGGTGCAGCCGGCGGGGACT-3'		
HRE1-mut	Forward	5'-GGGGACTTTGGAAACAATGTGTCACTTAGAATTT-3'		
	Reverse	5'-AAATTCTAAGTGACACATTGTTTCCAAAGTCCCC-3'		
HRE2-mut	Forward	5'-CCACCCTTCAATATTGGGTTACTTGGAAG-3'		
	Reverse	5'-CTTCCAAGTAACCCAATATTGAAGGGTGG-3'		
HRE3-mut	Forward	5'-GCACTTTACTACCAATCAAAGGTGAAAAC-3'		
	Reverse	5'-GTTTTCACCTTTGATTGGTAGTAAAGTGC-3'		
HRE4-mut	Forward	5'-GAAAAACCTTACTAATTGAGCATTTTCTT-3'		
	Reverse	5'-AAGAAAATGCTCAATTAGTAAGGTTTTTC-3'		
pGL3-hLRRK2-Luc	Forward	5'-GGTACCGATGGCTAACCCCTCTTTCC-3'		AC079630
	Reverse	5'-CTCGAGGTGGCACCTGCTTCCAAC-3'		
Primers used in ChIP analysis (Fig. 4f)				
mLRRK2 promoter-HRE3	Forward	5'-GGTTACTTGGAAGCAAATCC-3'	232	AC099704
	Reverse	5'-GATGGGTAAGAGGTGGAGG-3'		

[#]Sequences for the restriction enzymes BamHI and EcoRV, with an extra *g* added to improve digestion efficiency, are underlined and in italics.
